# Strong Inhibitory Activity and Action Modes of Synthetic Maslinic Acid Derivative on Highly Pathogenic Coronaviruses: COVID-19 Drug Candidate

**DOI:** 10.3390/pathogens10050623

**Published:** 2021-05-19

**Authors:** Raya Soltane, Amani Chrouda, Ahmed Mostafa, Ahmed A. Al-Karmalawy, Karim Chouaïb, Abdelwaheb dhahri, Rami Adel Pashameah, Ahlam Alasiri, Omnia Kutkat, Mahmoud Shehata, Hichem Ben Jannet, Jawhar Gharbi, Mohamed A. Ali

**Affiliations:** 1 Department of Basic Sciences, Adham University College, Umm Al-Qura University, Adham 21971, Saudi Arabia; rasoltan@uqu.edu.sa (R.S.); rapasha@uqu.edu.sa (R.A.P.); ajasiri@uqu.edu.sa (A.A.); 2 Faculty of Sciences, Tunis El Manar University, Tunis 1068, Tunisia; 3 Department of Chemistry, College of Science Al-Zulfi, Majmaah University, Al-Majmaah 11952, Saudi Arabia; 4 Laboratory of Interfaces and Advanced Materials, Faculty of Sciences, Monastir University, Monastir 5000, Tunisia; 5 Institute of Analytical Sciences, UMR CNRS-UCBL-ENS 5280, 5 Rue la Doua, CEDEX 09, 69100 Villeurbanne, France; 6 Center of Scientific Excellence for Influenza Viruses, National Research Centre, Dokki, 12622 Cairo, Egypt; omniakutkat@gmail.com (O.K.); Shehata_mmm@hotmail.com (M.S.); Mohamedahmedali2004@yahoo.com (M.A.A.); 7 Department of Pharmaceutical Medicinal Chemistry, Faculty of Pharmacy, Horus University-Egypt, 34518 New Damietta, Egypt; ahmed.alkarmalawy2019@gmail.com; 8 Laboratory of Heterocyclic Chemistry, Faculty of Science of Monastir, University of Monastir, Natural Products and Reactivity (LR11ES39), Team: Medicinal Chemistry and Natural Products, Avenue of Environment, Monastir 5019, Tunisia; karimchouaib1@hotmail.fr (K.C.); hichem.bjannet@gmail.com (H.B.J.); 9 Polymer Materials Engineering, University of Lyon, UMR CNRS 5223, Lyon, 69100 Villeurbanne, France; abdelwaheb.dhahri@gmail.com; 10 Department of Biological Sciences, College of Science, King Faisal University, Al-Ahsa 31982, Saudi Arabia; jagharbi@kfu.edu.sa

**Keywords:** maslinic acid, oleanolic acid, SARS-CoV-2, MERS-CoV, molecular docking, SAR

## Abstract

In late December 2019, a novel coronavirus, namely severe acute respiratory syndrome coronavirus 2 (SARS-CoV-2), escaped the animal–human interface and emerged as an ongoing global pandemic with severe flu-like illness, commonly known as coronavirus disease 2019 (COVID-19). In this study, a molecular docking study was carried out for seventeen (**17**) structural analogues prepared from natural maslinic and oleanolic acids, screened against SARS-CoV-2 main protease. Furthermore, we experimentally validated the virtual data by measuring the half-maximal cytotoxic and inhibitory concentrations of each compound. Interestingly, the chlorinated isoxazole linked maslinic acid (compound **17**) showed promising antiviral activity at micromolar non-toxic concentrations. Thoughtfully, we showed that compound **17** mainly impairs the viral replication of SARS-CoV-2. Furthermore, a very promising SAR study for the examined compounds was concluded, which could be used by medicinal chemists in the near future for the design and synthesis of potential anti-SARS-CoV-2 candidates. Our results could be very promising for performing further additional in vitro and in vivo studies on the tested compound (**17**) before further licensing for COVID-19 treatment.

## 1. Introduction

Since its emergence, coronavirus disease 2019 (COVID-19) has triggered a scary and contagious global pandemic of severe pneumonia-like disease, which causes respiratory illness and acute respiratory distress syndrome in human. Up to now, the race is still on to discover specific and efficient anti-viral drugs against SARS-CoV-2 to control COVID-19 to minimize its awful spread and transmission [1]. To this point, researchers have tried to make use of the existing anti-viral drugs that might combat the infection based on in silico predictions, or based on previous knowledge of other coronaviruses such as SARS-CoV and MERS-CoV. Therefore, a lot of FDA approved drugs have been tested or repositioned to act as anti-SARS-CoV-2 [2,3]. In parallel, several trials to virtually recommend or practically test the anti-SARS-CoV-2 activity of synthetic compounds, natural extracts and immunomodulatory agents have been attempted [4,5,6,7].

Triterpenes are a class of natural molecules made up of 30 carbon atoms. Biosynthetically, the 200 different skeletons known to date and isolated from natural sources come from the cyclization of (3S)-2,3-epoxy-2,3-dihydrosqualene and sometimes of squalene itself. These molecules are in most cases hydroxylated at C-3 position due to the opening of the epoxide during cyclization [8]. In the plant kingdom, terpenoids are secondary metabolites, whose ecological role has been proven, especially in the process of communications and defense. Triterpenes, of which more than 30,000 structures were previously isolated and characterized, is derived from natural sources or enzymatic reactions. Additionally, they comprise 47 sub-classes with enormous chemical diversity [8,9,10,11].

**Figure 1:**
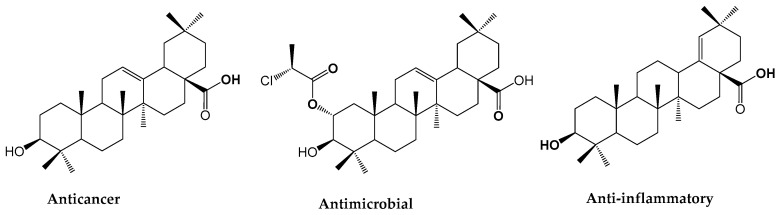
Examples of bioactive triterpenes.

Triterpenes are among the natural substances with promising biological, therapeutic and industrial value. In recent years, due to the broad spectrum of their biological potentials, triterpenes have been the subject of an intensive number of research works, mainly focused on their biopharmaceutical development. Indeed, triterpenes and their structural analogues display very varied biological effects such as hemolytic, hypocholesterolimic, immunostimulant, anti-inflammatory, antimicrobial, antiparasitic, cytotoxic, and anti-ulcer [12,13,14,15,16,17,18]. Moreover, triterpenes are known for their many important antiviral activities such as anti-hepatitis B virus activity, anti-HIV1 and 2, AIDS, and (to remove) anti-hepatitis C virus activity, and anti-Herpes simplex virus activity (Figure 1) [11,19].

These compounds and their antiviral activity have seriously attracted the attention of organic chemists, who have thought of chemically modifying them in different ways in order to improve their activities. Some of these analogues are employed as medicinal drugs to treat liver-related diseases [20].

Oleanolic acid (OA, **1**) and maslinic acid (MA, **2**) are triterpenoids isolated from several plants; in particular, from *Olea europaea* L. that possesses a variety of interesting biological activities. OA displays several biological potentials including antimicrobial, anticancer, and anti-inflammatory effects [19,21,22]. It has been found to inhibit the entry of several viruses such as HIV1 replication in acutely infected H9 lymphocyte cells [21,22,23]. In recent decades, OA has undergone several chemical modifications in order to synthesize new structural analogues with promising biological activities. These chemical modifications mainly concerned the C-3 alcohol and C-17 carboxylic acid functions as well as the C12-C13 unsaturation. Various OA congeners have shown antibacterial, anti-acetylcholinesterase, anticancer, anti-inflammatory, and anti-influenza virus activities and were found to be more active than OA [11,15,17,18,21,23,24,25,26,27].

Maslinic acid (MA) has shown a broad spectrum of biological activity. Indeed, it has been found to be inhibit glycogen phosphorylases [28] and protein tyrosine phosphatase 1B [29]. It also exhibited anti-inflammatory [20], antitumor [30], and anti-HIV-1 activities [31]. Several other interesting biological activities were also reported [15,17,18]. This triterpene acid has attracted the attention of several chemists, who have taken advantage of its various chemical functions to synthesize new series of structural analogues in order to improve some of their activities and also to understand the structure–activity relationship; we cite the example of esters, 3,5-isoxazoles and 1,5- and 1,4-disubstituted triazoles [15,17].

**Figure 2:**
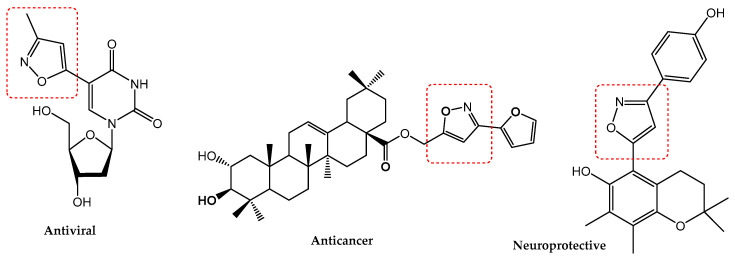
Previously reported bioactive isoxazoles.

Compounds bearing isoxazole moiety are of high importance due to their broad spectrum of biological properties including antiviral [32], antidiabetic [33], neuroprotective [34], anticancer and anti-inflammatory activities (Figure 2) [17,35].

**Figure 3:**
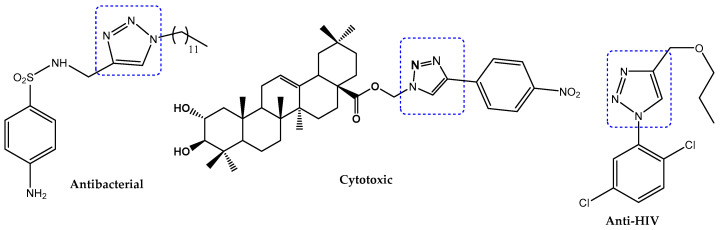
Previously reported bioactive triazoles.

1,2,3-Triazoles, constituting another important class of heterocycles, widely used in medicinal chemistry, act as pharmacophores and linkers between two or more substances of interest in molecular hybridization approaches [36]. Several of these triazoles have a wide spectrum of biological effects such as anti-HIV [37], antiviral [38], cytotoxic, anti-inflammatory [16,18], and antibacterial [39] activities (Figure 3).

In our previous works, Chouaïb et al. have described the isolation of OA and MA from pomace olive (*Olea europaea* L.) which have been used as a starting material to prepare new series of congeners constituting a library of novel pentacyclic triterpenoid derivatives bearing esters, 3,5-disubstituted isoxazoles, 1,5- and 1,4-disubstituted triazoles and were synthesized as antimicrobial, anti-inflammatory and anticancer agents [15,16,17,18]. In order to broaden the range of our biological tests on these derivatives taking into account the antiviral effect of oleanolic and maslinic acids as well as of isoxazoles and triazoles, molecular docking studies of OA, MA and most previously synthesized derivatives has been carried out targeting SARS-CoV-2 main protease which allowed one to select seventeen compounds to be evaluated for their antiviral activity against hCoV-19/Egypt/NRC-03/2020 (Accession Number on GSAID: EPI_ISL_430820) “NRC-03-nhCoV” virus [40].

Molecular docking is an important method of computational studies which are very promising tools in the new process of drug discovery and development as well [39]. Concerning the aforementioned crucial rule of main protease (M^pro^) for SARS-CoV-2 maturation, and in continuation to our previous work targeting SARS-CoV-2 [2,3,41,42,43,44], we decided to target SARS-CoV-2 M^pro^ using molecular docking studies and we further validated the antiviral activity of the 17 compounds representing OA, MA and their derivatives. Moreover, we further evaluated the impact of promising anti-SARS-CoV-2 compounds on different stages of the viral replication cycle.

## 2. Results and Discussion

Respiratory viruses represent a major public health concern, as they are highly mutated, resulting in the emergence of new strains with high pathogenicity. Currently, the whole world is suffering from a new evolving severe acute respiratory syndrome coronavirus 2 (SARS-CoV-2), which originally spread from the Hubei region, concisely in Wuhan City in early December 2019, and subsequently spread globally. By the end of January, and due to the increasing rate of SARS-CoV-2-infected confirmed cases associated with relatively high mortality rate, the WHO raised awareness and declared an emergency, claiming a potentially pandemic lethal infectious disease named COVID-19.

Initially, SARS-CoV-2 was considered as a zoonotic disease, based on a high number of infected people exposed to a wet animal market in the Wuhan Chinese city; however, transmission from person to person was confirmed, making the situation even more harsh. This condition resulted in many put quarantines in place, cancelling flights and suspending travel, with the consequence of huge economic burden.

The perceived threat of this pandemic has led many researchers and academic institutions all over the world to dissect COVID-19 from different perspectives, including but not limited to the route of virus transmission, diagnosis, virus inhibition treatments, and vaccine preparation. Apparently, no treatment or vaccine until now has been discovered; nevertheless, the reposition of the currently approved antiviral drugs are in use. Special consideration for more susceptible people such as elderly people, patients with chronic diseases, and health care takers should be applied. This study is considered as a serious attempt to synthesize a unique series of oleanolic acid (**1**) and maslinic acid (**2**) derivatives as anti-SARS-CoV-2 candidates.

### 2.1. Chemistry

#### 2.1.1. Green Chemistry Extraction of Compounds **1** and **2**

In this study, the ultrasound-assisted extraction of triterpenoids **1** and **2** from pomace olive of *Olea europaea* L. was optimized compared to previous methods used. The triterpenoid yield was 3.6 and 9.2 mg/g dry weight (DW), respectively, under optimal conditions. As shown in Table 1, the present study is the first report on the simultaneous extraction of triterpenoids **1** and **2** from pomace olive (*Olea europaea* L.) cultivar: Chemlali with a large amount under green chemistry conditions (ultrasonic-assisted extractions, then centrifugation, and purification), compared with those of previous works. This developed process could be useful in the preparation of a triterpenoid-rich ingredient that holds great promise for application in the therapeutic industry.

#### 2.1.2. Synthesis

**Scheme 1:**
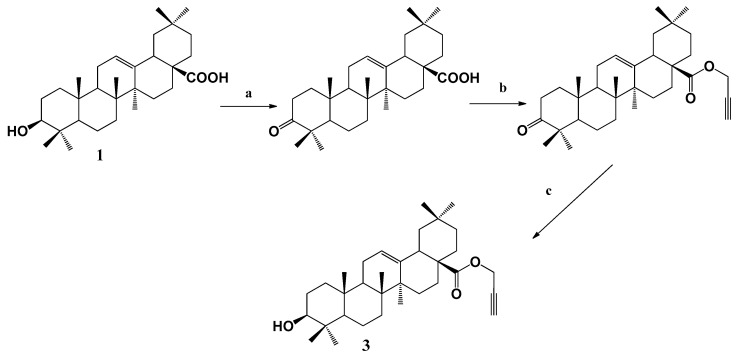
Propargylation of oleanolic acid (**1**). Reagents and conditions: (a) Jones oxidation (CrO_3_, H_2_SO_4_/acetone), 0 °C (b) propargyl bromide, NaH, dry DMF, rt (c) NaBH_4_, CH_3_OH/THF, Microwave (250 W, 3 min).

To prepare the propargylated ester **3**, derived from oleanolic acid **1**, we started by the protection of the hydroxyl group at C-3 via its oxidation in order to direct the propargylation towards the carboxylic acid function in C-17 (Scheme 1). Then, we subjected the oxidized intermediate to the propargylation in dry *N*,*N*-Dimethylformamide (DMF) at room temperature in the presence of NaH and propargyl bromide for 2 h, yielding the desired propargylated intermediate which was reduced with sodium borohydride under microwave irradiation, to obtain the propargyl-(3β)-3-hydroxyolean-12-en-28-oate (**3**).

Similarly, maslinic acid **2** was subjected to the same reaction of propargylation, without oxidation beforehand, yielding a mixture of differently propargylated compounds which were separated over silica gel column chromatography to afford compounds **4–7** (Table 2) [16,17].

**Scheme 2:**
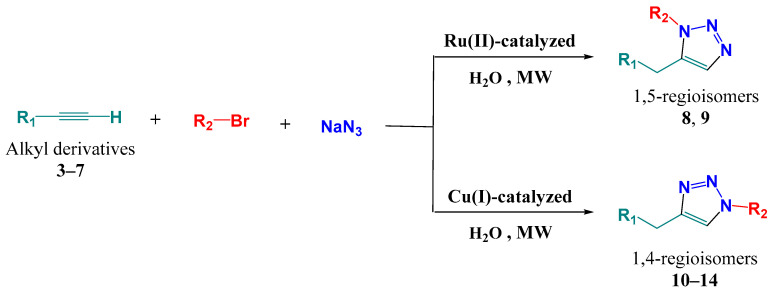
Synthesis of the 1,4- and 1,5-triazolyl (**8–14**) derivatives.

A regiospecific, facile and practical multicomponent synthesis of 1,5-triazolyl derivatives (**8** and **9**) by Ru(II)-catalyzed, and mono-, bis- and tri-1,4-triazolyl derivatives (**10–14**) by Cu(I)-catalyzed was carried out (Scheme 2).

**Scheme 3:**
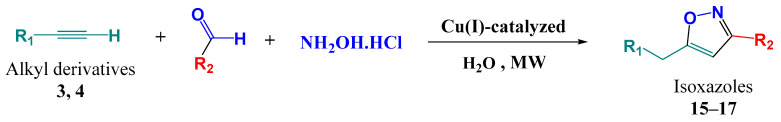
Synthesis of the 3,5-disubstituted isoxazol (**15–17**).

According to the same method as mentioned above, the dipolarophiles **3** and **4** were found to be readily cyclized to the 3,5-disubstituted isoxazoles (**15–17**) (Scheme 3). In fact, copper-catalyzed microwave-assisted multicomponent 1,3-dipolar cycloaddition between pentacyclic triterpenoid alkyne derivatives **3** and **4**, and the appropriate aldehyde, regiospecifically afforded 3,5-disubstituted isoxazoles (**15–17**) in quantitative yields (Table 2).

All the reactions were assisted by microwave irradiation avoiding toxic reagents and solvents. The products were obtained from the reaction mixture by simple purification in almost quantitative yields (Table 2).

### 2.2. Anti-SARS-CoV-2 Activity

#### 2.2.1. Molecular Docking Studies

By studying the binding pocket of the SARS-CoV-2M^pro^, it was found that the co-crystallized native inhibitor (N3) fitted inside its binding pocket of the main protease receptor asymmetrically. N3 is a designed inhibitor for SARS-CoV-2 M^pro^ that was built from amino acids based on the M^pro^ pocket amino acids and cannot used medicinally (Table 2).

Molecular docking of oleanolic acid (**1**), maslinic acid (**2**), and other oleanolic acid and maslinic acid derivatives (**3–17**) (Table 2), and N3 inhibitor (**18**) into the main protease binding site was performed. Almost all the tested compounds (**1–17**) were stabilized inside the M^pro^ binding site by variable scores and binding interactions with the amino acids of the receptor pocket. It is worth mentioning that, especially for both compounds **17** and **3**, the obtained binding scores and modes were greatly similar to that of the docked N3 inhibitor (**18**), as depicted in Table 3. Additionally, the 2 D binding modes of the tested compounds (**1–17**) with the amino acids of the SARS-CoV-2 M^pro^ pocket are presented in Supplementary Table S2. All of the tested compounds (**1–17**) achieved promising binding scores (from −6.55 to −10.20 kcal/mol), compared to the docked co-crystallized N3 inhibitor (**18**) with a binding score of −9.70 kcal/mol. The RMSD_refine values of the selected poses were within the acceptable range (from 0.9 to 2.25).

The docked N3 inhibitor (**18**) was stabilized inside the SARS-CoV-2 M^pro^ pocket through a hydrogen bond formation with Thr26 amino acid at 3.08 A°. Additionally, the most promising cytotoxic compound (**17**) fitted inside the binding site of the SARS-CoV-2 M^pro^ by three hydrogen bond formations, two of them with Thr24 at 2.71 and 3.19 A°, respectively, and the third one was formed with Thr26 at 3.28 A°. Moreover, compound (**3**) formed three hydrogen bonds with the previously mentioned two amino acids as well. It formed two hydrogen bonds with Thr24 at 2.80 and 3.18 A°, respectively, and the third one with Thr26 at 3.37 A°. Additionally, it is worth mentioning that the achieved binding scores for compounds **17** and **3** were −8.00 and −7.55 kcal/mol, respectively, compared to the docked co-crystallized N3 inhibitor (**18**) (−9.70 kcal/mol) (Table 3).

By considering the docking findings of the selected tested compounds (**1–17**) against the binding pocket of the SARS-CoV-2 main protease compared to its co-crystallized inhibitor (N3) as a reference standard, and its great matching with the cytotoxicity studies applied, we can build a very promising and recommended idea about their mechanism of action.

#### 2.2.2. Compound **17** Showed Promising Anti-SARS-CoV-2 Activity at Non-Toxic Concentrations

**Figure 4:**
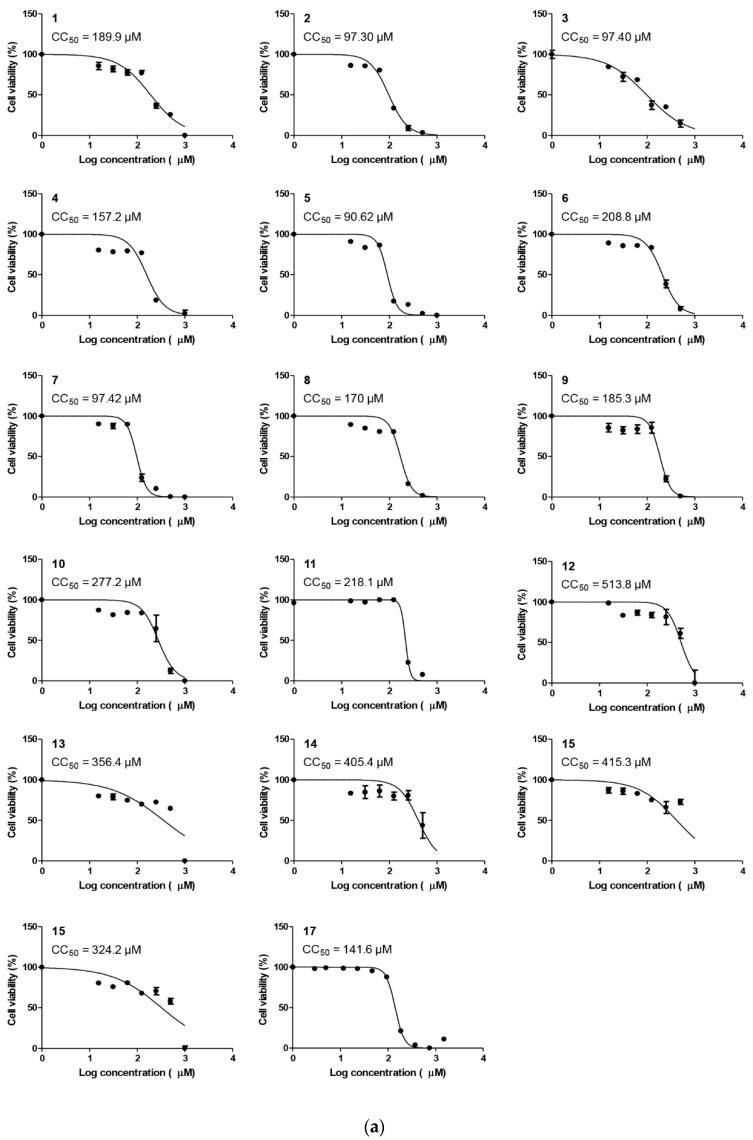

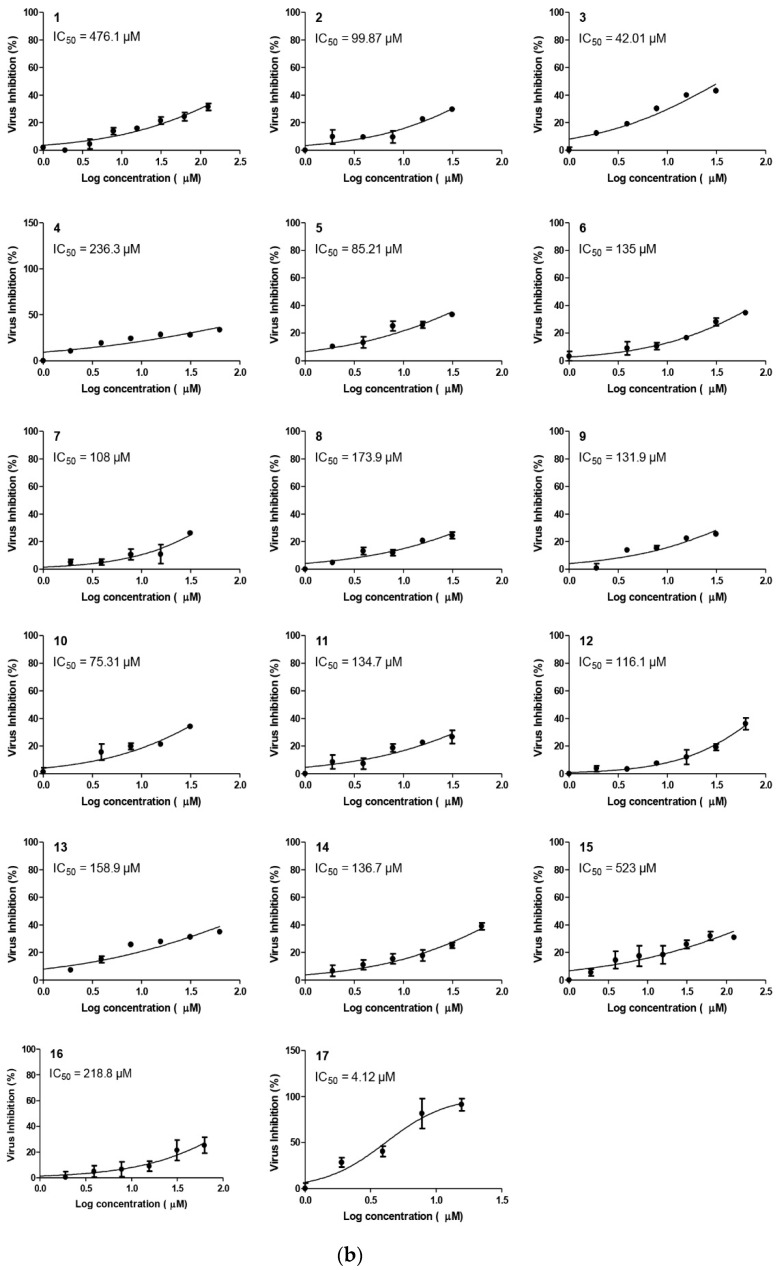
Anti-SARS-CoV-2 activities of tested compounds (**1–17**). (**a**) Half-maximal cytotoxic concentrations (CC_50_) on Vero E6 cells, and (**b**) half-maximal inhibitory concentrations (IC_50_) against NRC-03-nhCoV in Vero E6. Inhibitory concentration 50% (IC_50_) values were calculated using nonlinear regression analysis of GraphPad Prism software (version 5.01) by plotting log inhibitor versus normalized response (variable slope).

To identify the proper concentrations to define the antiviral activity of the selected compounds, half maximal cytotoxic concentration “CC_50_” was calculated for each individual drug (Figure 4). The antiviral screening revealed that one compound “**17**” out of the tested panel compounds exhibits a promising in vitro activity against SARS-CoV-2 “NRC-03-nhCoV” (IC_50_ = 4.12 µM) with a satisfactory selectivity index (≥7.5) (Figure 4 and Table 4).

#### 2.2.3. Compound **17** Showed Promising Anti-MERS-CoV Activity and Non-Toxic Concentrations

**Figure 5:**
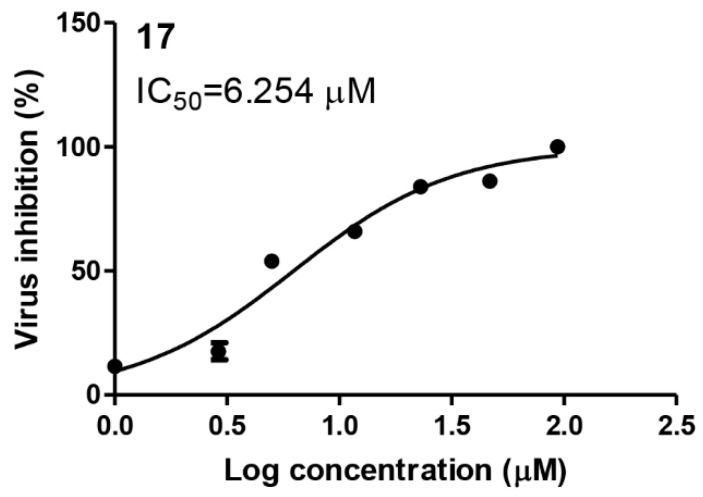
Half-maximal inhibitory concentrations (IC_50_) against Middle East respiratory syndrome-related coronavirus isolate NRCE-HKU270 (accession number: KJ477103.2; NRCE-HKU270 is obtained from CSEIVs) [45] in Vero E6 cells using crystal violet assay. Inhibitory concentration 50% (IC_50_) values were calculated using nonlinear regression analysis of GraphPad Prism software (version 5.01) by plotting log inhibitor versus normalized response (variable slope).

To assess the antiviral activity of compound **17** against other highly pathogenic coronaviruses, we assayed its activity against MERS-CoV virus “NRCE-HKU270”. Interestingly, compound **17** showed promising antiviral activity against NRCE-HKU270 at micromolar concentrations (Figure 5).

#### 2.2.4. Structure–Activity Relationship (SAR) Study

**Figure 6:**
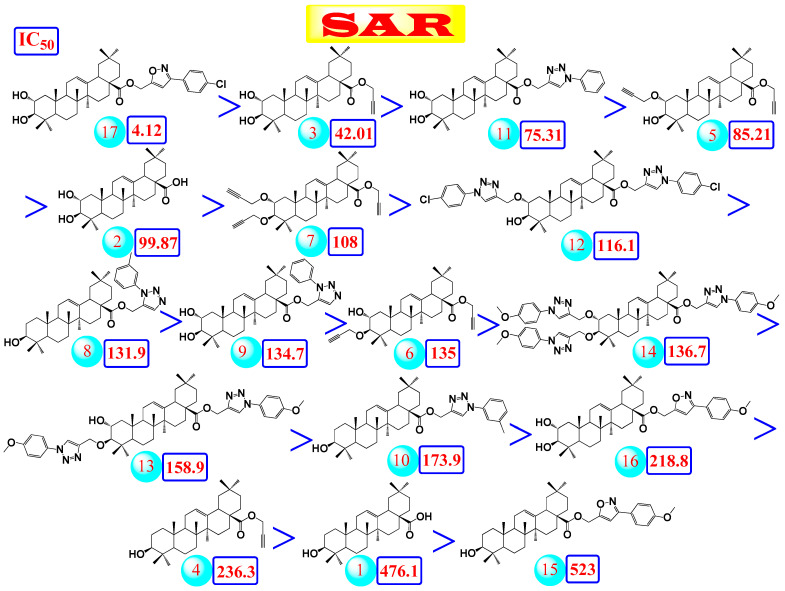
Structure–activity relationship (SAR) of the selected tested compounds (**1–17**) based on both their cytotoxic and docking studies against SARS-CoV-2.

By studying the relations between the structures of the selected tested compounds (**1–17**) and their cytotoxic IC_50_ values (Figure 6) and using the molecular docking results as a way to relate their activities to their binding affinities as SARS-CoV-2 main protease inhibitors, we may conclude that;

(a) The common shared steroidal nucleus of the selected tested compounds (**1–17**) appeared to be very promising for their antiviral activities. Molecular docking revealed the great stabilization of the tested compounds inside the branched large pocket of SARS-CoV-2 main protease as a proposed mechanism of action.
(b) The presence of OH groups at positions 2 and 3 of the steroidal nucleus seems to be very crucial for the antiviral activity. Most compounds (**17**, **3**, and **10**) containing both 2- and 3-OH groups achieved high to moderate activities (4.12–99.87 µM). Docking studies referred to the involvement of both OH groups in hydrogen bond formations with Thr24 and/or Thr26 amino acids of the binding pocket.
(c) Compound (**17**) containing the isoxazole side chain with a p-chlorophenyl substitution (an electron withdrawing lipophilic group) achieved a very high cytotoxic activity (IC_50_ = 4.12 µM). However, both compounds (**16** and **15**) containing the isoxazole side chain with a p-methoxyphenyl substitution (an electron donating hydrophilic group) achieved low cytotoxic activities (IC_50_ = 218.8 and 523 µM, respectively). Therefore, the lipophilic p-chloro group is very important for the antiviral activity. Again, compound (**16**) containing both 2- and 3-OH groups showed a higher cytotoxic activity compared to compound (**15**) with only 3-OH group as mentioned before.
(d) The presence of the acetyl methylene group at positions 2 and/or 3 in compounds (**5**, **7**, and **6**) gave better cytotoxic activities compared to the heteroaromatic ring substitutions at the same positions in compounds (**12**, **14**, and **13**).
(e) The cytotoxic activity of compound (**17**) containing the isoxazole side chain with a p-chlorophenyl substitution (IC_50_ = 4.12 µM) was clearly higher than the respective same compound (**10**) with a phenyl triazole side chain instead (IC_50_ = 75.31 µM).

Collectively, these studies greatly suggested the very promising affinities of the selected tested compounds (**1–17**) against SARS-CoV-2, especially compounds **17** and **3**. Accordingly, we recommend such compounds for more advanced in vitro and in vivo studies to obtain an effective therapeutic against the pandemic SARS-CoV-2. Moreover, the aforementioned studied compounds could be used alone or in combinations with each other’s against SARS-CoV-2.

#### 2.2.5. Mechanism of Actions

**Figure 7:**
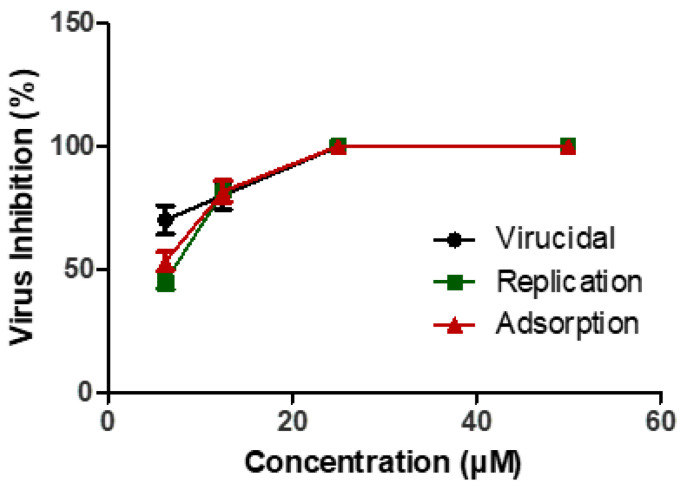
Mode of antiviral action of compound **17** against SARS-CoV-2 in Vero E6 cells as measured by plaque reduction assay. Inhibitory percent values were calculated and plotted against inhibitor concentration using GraphPad Prism software (version 5.01).

The virucidal effect of the antiviral compound **17** against SARS-CoV-2 virus has been studied, and interference with viral adsorption and replication was demonstrated. Besides the virucidal effect of compound **17**, it could efficiently impair viral replication and adsorption (Figure 7).

## 3. Materials and Methods

### 3.1. Chemistry

#### 3.1.1. Green Chemistry Condition for Extraction and Purification of OA (**1**) and MA (**2**) from the Pomace Olive of *Olea europaea* L.

Using cultivar Chemlali pomace olive resulting from olive fruit pressing as a natural substance, the most suitable conditions to carry out the extraction and purification of compounds **1** and **2** were explored. The optimal conditions were determined to be the ultrasonic power of 250 W, extraction temperature of 60 °C, ratio of liquid to solid of 30 mL/g, and 60 min extraction time, using aqueous ethanol (concentration of 70%) as the extracting solvent. After the evaporation of the ethanol under reduced pressure, the extract was precipitated at +4 °C for 48 h. After filtration, the extract was centrifuged at 5000 rpm/5 min, yielding a mixture of compounds **1** and **2** according to TLC analysis (13.5% (*w/w*)).The crude mixture was recrystallized from the mixture EtOH/AcOEt (8:2, *v/v*) to obtain pure maslinic acid **2** (9.2 mg/g DW). The filtrate was concentrated and recrystallized from ethanol to obtain pure oleanolic acid **1** (3.6 mg/g DW).

#### 3.1.2. Synthesis

##### Multicomponent Synthesis of 1,5-Disubstituted Triazoles in Water Catalyzed by Cp*RuCl (PPh_3_)_2_

To a solution of alkyne **3** or **4** (0.1 g) and NaN_3_ (1.1 mmol) in water, Cp*RuCl(PPh_3_)_2_ (5 mol%) was added at room temperature. To this mixture, alkyl halide (1.0 mmol) was added, and the reaction mixture was subjected to microwave irradiations at 250 W until total conversion of the starting alkyne (TLC). The crude mixture was extracted with EtOAc (3 × 30 mL). The organic layer was dried over Na_2_SO_4_. After the removal of solvent under reduced pressure, the resulting residue was purified by recrystallization from ethanol, to afford the desired products **8** and **9** in 90% and 92% yields, respectively [46,47].

##### Cu(I))-Cuatalyzed Multicomponent Huisgen 1,3-Dipolar Cycloaddition Reaction

To a suspension of the corresponding terminal alkyne (100 mg) and NaN_3_ (1.1 mmol) in water (3 mL), the corresponding alkyl halide (1.0 mmol) was added, followed by the addition of the CuI catalyst (0.1 equiv) (Scheme 1). The reaction mixture was subjected to microwave irradiations at 250 W, which was completed within 4–6 min (TLC). The crude mixture was extracted with EtOAc (3 × 30 mL) and the combined organic layer was dried over sodium sulfate, concentrated under reduced pressure and purified through recrystallization from ethanol, to give pure 1,4-triazolyl derivatives **10–14** in 90–98% yields [48].

##### Multicomponent Synthesis of 3,5-Disubstituted Isoxazoles in Water Catalyzed by CuI

At room temperature and stirring, to a mixture of alkyl derivatives **3** or **4** (0.1 g), NH_2_OH.HCl (1.2 equiv) and 0.1 equiv copper(I) iodide (CuI) in water, the appropriate aldehyde (1.1 equiv) was added, and the mixture was subjected to microwave irradiations at 250 W for 5–10 min (Scheme 2). The crude mixture was diluted with water and then extracted with EtOAc (3 × 30 mL). The organic layer was dried over Na_2_SO_4_. After the removal of solvent in vacuo, the resulting residue was purified by recrystallization from ethanol, to obtain the desired products **15–17** in 98, 87 and 96% yield, respectively [49].

The spectral data of all the synthesized compounds are in agreement with the literature data [16,17,18,50].

### 3.2. Biological

#### 3.2.1. Molecular Docking Studies

Molecular docking studies using MOE 2019.012 suite [51] were performed to propose the expected mechanism of action for the 17 tested compounds as SARS-CoV-2 M^pro^ inhibitors through evaluating their binding scores and interaction modes at the SARS-CoV-2 M^pro^ receptor active site. The co-crystallized native inhibitor (N3) was extracted from the protein complex and inserted in our calculations as a reference standard.

#### 3.2.2. Preparation of the Selected Tested Compounds

Each member of the previously mentioned selected compounds (**1–17**) was sketched using the ChemDraw program, transferred to MOE, and then subjected to the preparation process for the docking step as described earlier [52]. Then, all the prepared compounds (**1–17**) were imported in one database together with the co-crystallized native inhibitor (N3, 18) and saved as an MDB file to be used during the docking procedure.

#### 3.2.3. Preparation of the Active Site of the Applied SARS-CoV-2 M^pro^ Receptor

The Protein Data Bank website was used to extract the X-ray structure of the SARS-CoV-2 M^pro^ receptor (PDB ID: 6LU7) [53]. Then, all default steps for its preparation were run as described earlier in detail [54,55].

#### 3.2.4. Validation of the Applied MOE Program

Before applying the docking process, we first performed a validation process for the prepared target SARS-CoV-2 M^pro^ receptor by redocking its co-crystallized native inhibitor (N3) inside its active site and comparing the resulted RMSD values between the co-crystallized (native) and docked N3 molecules. The obtained low RMSD value (1.20) indicated a promised valid performance (Supplementary Table S1) [56].

#### 3.2.5. Docking of the Prepared Database to the Binding Pocket of SARS-CoV-2 M^pro^ Receptor

The previously discussed database was selected and inserted at the site of ligand at the beginning of the general docking process and the default methodology was performed as described before [57]. Finally, we selected the best poses according to their binding scores, RMSD values, and binding modes for further considerations.

#### 3.2.6. In Vitro Antiviral Bioassay

##### Cytotoxicity (CC_50_) Determination

To assess the half maximal cytotoxic concentration (CC_50_), stock solutions of the test compounds were prepared in 10% DMSO in ddH_2_O and diluted further to the working solutions with DMEM. The cytotoxic activity of the extracts was tested in VERO-E6 cells by using crystal violet assay as previously described [58,59] with minor modifications. Briefly, the cells were seeded in 96-well plates (100 µL/well at a density of 3 × 105 cells/mL) and incubated for 24 h at 37 °C in a humidified 5% CO_2_ incubator. After 24 h, cells were treated with various concentrations of the tested compounds in triplicates. Seventy two hours later, the supernatant was discarded, and cell monolayers were fixed with 10% formaldehyde for 1 h at room temperature (RT). The fixed monolayers were then dried and stained with 50 µL of 0.1% crystal violet for 20 min on a bench rocker at RT. The monolayers were then washed, dried and the crystal violet dye in each well was then dissolved with 200 µL of methanol for 20 min on a bench rocker at RT. The absorbance of crystal violet solutions was measured at λmax 570 nm as a reference wavelength using a multi-well plate reader. Cytotoxic concentration 50% (CC_50_) values were calculated using nonlinear regression analysis of GraphPad Prism software (version 5.01) by plotting log inhibitor versus normalized response (variable slope).

##### Inhibitory Concentration 50 (IC_50_) Determination

The IC_50_ values for the tested compounds were determined as previously described [2,3], with minor modifications. Briefly, in 96-well tissue culture plates, 2.4 × 10^4^ Vero-E6 cells were distributed in each well and incubated overnight at a humidified 37°C incubator under 5% CO_2_ condition. The cell monolayers were then washed once with 1× PBS. An aliquot of the SARS-CoV-2 “NRC-03-nhCoV” virus [40] containing 100 TCID50 was incubated with serial diluted compounds and kept at 37 °C for 1 h. The Vero-E6 cells were treated with virus/compound mix and co-incubated at 37 °C in a total volume of 200 µL per well. Untreated cells infected with virus represented the virus control; however, cells that were not treated and not infected were the cell control. Following incubation at 37 °C in 5% CO_2_ incubator for 72 h, the cells were fixed with 100 μL of 10% paraformaldehyde for 20 min and stained with 0.5% crystal violet in distilled water for 15 min at RT. The crystal violet dye was then dissolved using 100 μL of absolute methanol per well and the optical density of the color was measured at 570 nm using the Anthos Zenyth 200 rt plate reader (Anthos Labtec Instruments, Heerhugowaard, the Netherlands). The IC_50_ of the compound was that required to reduce the virus-induced cytopathic effect (CPE) by 50%, relative to the virus control.

##### Mechanism of Action(s) for Compound **17**

To investigate whether the tested compound with high selectivity index and antiviral activity against NRC-03-nhCoV affect (a) viral adsorption, (b) viral replication or (c) viricidal effect, a plaque infectivity reduction assay was performed according to the following protocols:

(a) Viral adsorption mechanism

The viral adsorption mechanism was assayed according to a protocol by Zhang et al. [60] with minor modifications. Vero-E6 cells were cultivated in a 6-well plate (10^5^ cells/mL) for 24 h at 37 °C. Each tested drug was applied in 500 µL of medium without supplements and co-incubated with the cells for 2 h at 4 °C. The non-absorbed drug in the applied inocula was removed by washing cells three times with 1x PBS. The NRC-03-nhCoV was diluted to a countable range “10^−4^” and co-incubated with the pretreated cells. About 1 h post-incubation, the inocula were removed and 3 mL of agarose overlay (1:1 mixture of warmed 2× plaque media and a stock solution of heated 2% agarose) were added. The plates were kept at room temperature to solidify and then incubated at 37 °C to allow the formation of viral plaques. The plaques were fixed using 10% formaldehyde, stained using crystal violet (1% crystal violet in 20% ethanol and distilled H_2_O), washed with rinse water and dried to calculate the percentage reduction in plaque formation compared to control wells, which comprised untreated Vero-E6 cells directly infected with NRC-03-nhCoV.

(b) Viral replication mechanism

The impact of the tested drug on viral replication was determined as previously described [50]. Vero-E6 cells were cultivated in a 6-well plate (10^5^ cell/mL) for 24 h at 37 °C. The NRC-03-nhCoV of the countable range was inoculated directly to the cells and incubated for 1 h at 37 °C. The inocula containing the non-adsorbed virus were removed by washing infected cell monolayers three times with 1× PBS. The analyzed compound was then applied in varying concentrations to infected cells for another 1 h contact time at 37 °C. After removing the inocula containing the tested drug, agarose overlay were added to the cells. The plates were kept at room temperature to solidify and then incubated at 37 °C to allow the formation of viral plaques. The plaques were fixed using 10% formaldehyde, stained using 1% crystal violet washed with rinse water and dried to calculate the percentage reduction in plaque formation compared to control wells, which comprised untreated Vero-E6 cells directly infected with NRC-03-nhCoV.

(c) Virucidal mechanism

The virucidal mechanism was assayed following a previously described protocol [61]. Briefly, Vero-E6 cells (105 cells/mL) were cultivated for 24 h at 37 °C in a 6-well plate. In an eppendorf, 200 µL of stock NRC-03-nhCoV were mixed with the tested compound at different concentrations. The mixture was kept for 1 h at room temperature against the control untreated virus. After 1 h of incubation, the mixture was ten-fold serial diluted four times using serum-free medium to obtain a countable range of the virus. An aliquot of 100 µL of each dilution were added to the Vero-E6 cell monolayer. After 1 h contact time, the agarose overlay was added to the cell monolayer. The plates were kept at room temperature to solidify and then incubated at 37 °C to allow the formation of viral plaques. The plaques were fixed using 10% formaldehyde, stained using 1% crystal violet washed with rinse water and dried to calculate the percentage reduction in plaque formation compared to control wells, which comprised untreated Vero-E6 cells directly infected with NRC-03-nhCoV.

## 4. Conclusions

Oleanolic acid (**1**) and maslinic acid (**2**), isolated from pomace olive oil, and a series of their synthesized structural analogues (**3–17**) were first evaluated using molecular docking for their proposed expected inhibitory activities against SARS-CoV-2 main protease, and then were screened for the same activity. Most of the tested compounds showed promising affinities towards SARS-CoV-2 main protease, especially for the most active member (**17**). Accordingly, we recommend such compounds for more advanced in vitro and in vivo studies to obtain an effective therapeutic against the pandemic SARS-CoV-2. Moreover, the aforementioned studied compounds could be used alone or in combinations with each other against SARS-CoV-2.

## 5. Patents

This work presented in this manuscript is submitted as a patent application to Saudi Authority for Intellectual Property under number 121420640.

## Figures and Tables

**Table 1:** Methods and extraction yield of oleanolic acid (**1**) and maslinic acid (**2**) from different pomace olive cultivars.

Cultivar	Extraction Methods	Yield of Extraction (mg/g DW)		References
		Oleanolic Acid 1	Maslinic Acid 2	
Picual	Solid–liquid extraction (maceration)	0.500	1.200	[41]
Hojiblanca		0.500	1.300	
Arbequina		0.400	1.500	
Non-indicated		0.015	0.034	[42]
Manzanilla	Solid–liquid extraction (centrifugation)	0.274	0.824	[43]
Hojiblanca		0.565	0.904	
Cacereña		0.185	0.295	
Kalamata		0.841	1.318	
Picual	Ultrasonic-assisted extraction	1.003	2.440	[44]
Kalamon		0.838	2.100	
Chemlali	Solid–liquid, then ultrasonic-assisted extractions	3.400	8.500	[15]
Chemlali	Ultrasonic-assisted extractions, then centrifugation isolation	3.6	9.2	This work

**Table 2:** Oleanolic acid (**1**), maslinic acid (**2**) and their derivatives **3–17** screened for their anti- SARS-CoV-2 activity in addition to N3 (the co-crystallized native inhibitor of SARS-CoV-2).

Code	Nomenclature	Chemical Structures	Yield (%)
**1**	Oleanolic acid	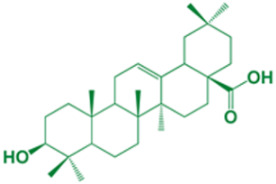	-
**2**	Maslinic acid	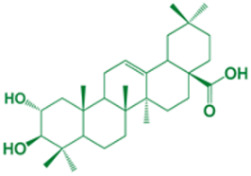	-
**3**	Propargyl-(3β)-3-hydroxyolean-12-en-28-oate	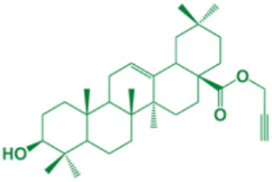	99
**4**	Propargyl-(2α,3β)-2,3-dihydroxyolean-12-en-28-oate	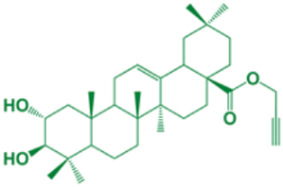	25
**5**	Propargyl-(2α)-2-(propargyloxy)-(3β)-3-hydroxy-olean-12-en-28-oate	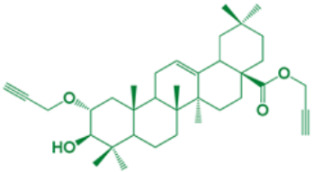	23
**6**	Propargyl-(3α)-3-(propargyloxy)-(2β)-2-hydroxy-olean-12-en-28-oate	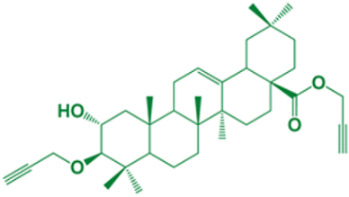	20
**7**	Propargyl-(2α,3β)-2,3-bis(propargyloxy)-olean-12-en-28-oate	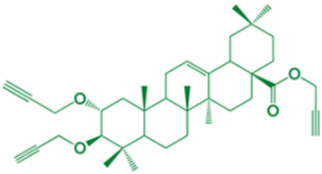	31
**8**	(1-(3-methylphenyl)-1H-1,2,3-triazol-5-yl)methyl-(3β)-3-hydroxyolean-12-en-28-oate	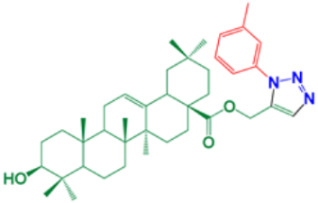	90
**9**	(1-(3-methylphenyl)-1H-1,2,3-triazol-5-yl)methyl-(2α,3β)-2,3-dihydroxyolean-12-en-28-oate	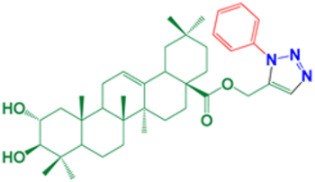	92
**10**	(1-(3-methylphenyl)-1H-1,2,3-triazol-4-yl)methyl-(3β)-3-hydroxyolean-12-en-28-oate	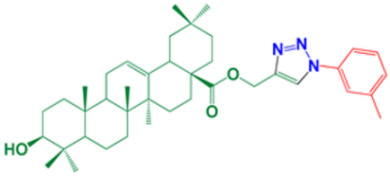	98
**11**	(1-Phenyl-1H-1,2,3-triazol-4-yl)methyl-(2α,3β)-2,3-dihydroxyolean-12-en-28-oate	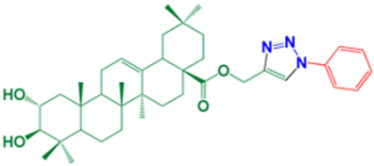	96
**12**	(1-(4-Chlorophenyl)-1H-1,2,3-triazol-4-yl)methyl-(2α,3β)-2-((1-(4-chlorophenyl)-1H-1,2,3-triazol-4-yl)methoxy)-3-hydroxyolean-12-en-28-oate	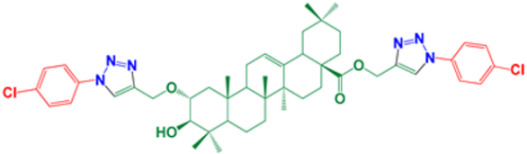	94
**13**	(1-(4-Methoxyphenyl)-1H-1,2,3-triazol-4-yl)methyl-(2α,3β)-2-hydroxy-3-((1-(4-methoxyphenyl)-1H-1,2,3-triazol-4-yl)methoxy)-olean-12-en-28-oate	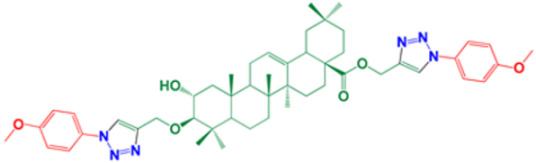	96
**14**	(1-(4-Methoxyphenyl)-1H-1,2,3-triazol-4-yl)methyl-(2α,3β)-2,3-bis((1-(4-methoxyphenyl)-1H-1,2,3-triazol-4-yl)methoxy)-olean-12-en-28-oate	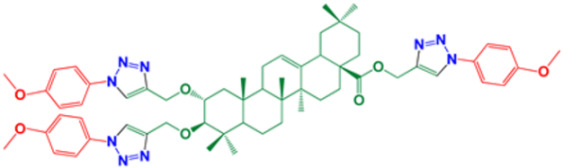	92
**15**	(3-(4-methoxyphenyl) isoxazol-5-yl) methyl-(3β)-3-hydroxyolean-12-en-28-oate	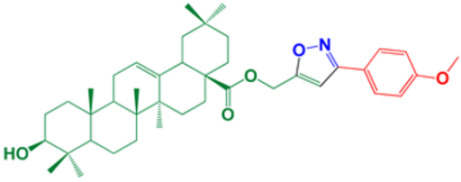	98
**16**	(3-(4-methoxyphenyl) isoxazol-5-yl) methyl-(2α,3β)-2,3-dihydroxyolean-12-en-28-oate	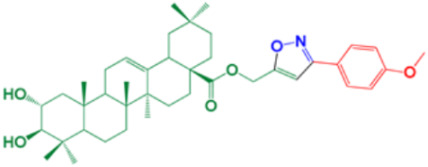	87
**17**	(3-(4-chlorophenyl) isoxazol-5-yl) methyl-(2α,3β)-2,3-dihydroxyolean-12-en-28-oate	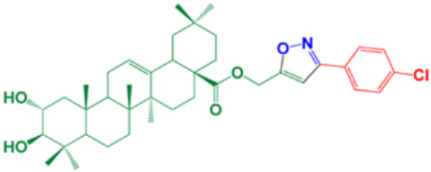	96
**18**	N3 (co-crystallized native inhibitor of SARS-CoV-2)	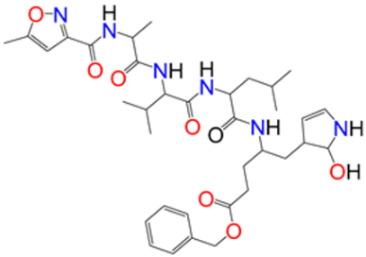	-

**Table 3:** 3D representations showing the binding interactions and positioning between the two promising tested compounds (**17** and **3**) and the N3-binding pocket in comparison to the docked N3 inhibitor (**18**).

Code	3D Binding Interactions	3D Pocket Positioning
**17**	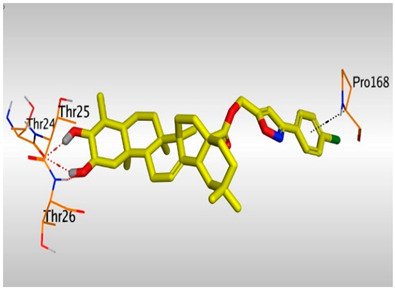	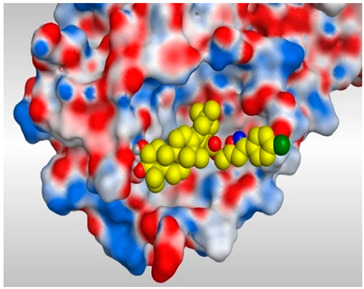
**3**	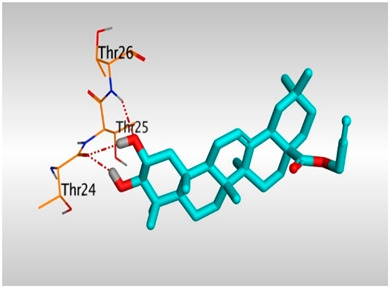	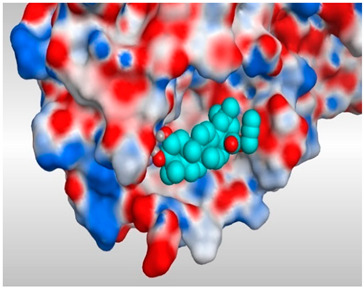
**N3 (18)**	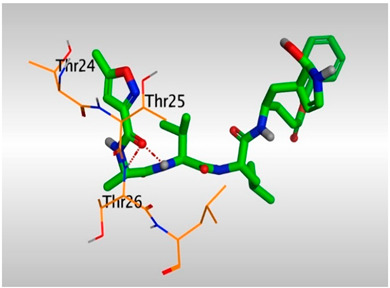	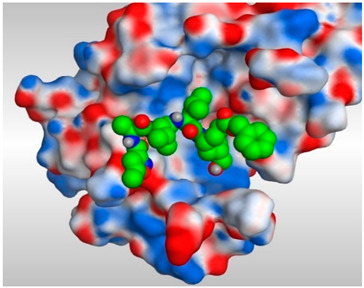

**Table 4:** Selectivity index of tested compounds **1–17**.

Compound	CC_50_	IC_50_	Selectivity Index (CC_50_/IC_50_)
**1**	189.9	476.1	0.39
**2**	97.30	99.87	0.97
**3**	97.40	42.01	2.32
**4**	157.2	236.3	0.67
**5**	90.62	85.21	1.06
**6**	208.8	135	1.55
**7**	97.42	108	0.90
**8**	170	173.9	0.98
**9**	185.3	131.9	1.40
**10**	277.2	75.31	3.68
**11**	218.1	134.7	1.62
**12**	513.8	116.1	4.42
**13**	356.4	158.9	2.24
**14**	405.4	136.7	2.96
**15**	415.3	523	0.79
**16**	324.2	218.6	1.48
**17**	141.6	4.12	34.36

## Data Availability

Not applicable.

## Supplementary Materials

The following are available online at https://www.mdpi.com/article/10.3390/pathogens10050623/s1.

## References

[1] Kumar S., Singh R., Kumari N., Karmakar S., Behera M., Siddiqui A.J., Rajput V.D., Minkina T., Bauddh K., Kumar N. (2021). Current understanding of the influence of environmental factors on sars-cov-2 transmission, persistence, and infectivity. Environ. Sci. Pollut. Res. Int..

[2] Mostafa A., Kandeil A., Elshaier Y.A.M.M., Kutkat O., Moatasim Y., Rashad A.A., Shehata M., Gomaa M.R., Mahrous N., Mahmoud S.H. (2020). Fda-approved drugs with potent in vitro antiviral activity against severe acute respiratory syndrome coronavirus 2. Pharmaceuticals.

[3] Alnajjar R., Mostafa A., Kandeil A., Al-Karmalawy A.A. (2020). Molecular docking, molecular dynamics, and in vitro studies reveal the potential of angiotensin ii receptor blockers to inhibit the covid-19 main protease. Heliyon.

[4] Mahmoud D.B., Shitu Z., Mostafa A. (2020). Drug repurposing of nitazoxanide: Can it be an effective therapy for covid-19?. J. Genet. Eng. Biotechnol..

[5] Surti M., Patel M., Adnan M., Moin A., Ashraf S.A., Siddiqui A.J., Snoussi M., Deshpande S., Reddy M.N. (2020). Ilimaquinone (marine sponge metabolite) as a novel inhibitor of sars-cov-2 key target proteins in comparison with suggested covid-19 drugs: Designing, docking and molecular dynamics simulation study. RSC Adv..

[6] Alzahrani F.A., Saadeldin I.M., Ahmad A., Kumar D., Azhar E.I., Siddiqui A.J., Kurdi B., Sajini A., Alrefaei A.F., Jahan S. (2020). The potential use of mesenchymal stem cells and their derived exosomes as immunomodulatory agents for covid-19 patients. Stem Cells Int..

[7] Fayed M.A.A., El-Behairy M.F., Abdallah I.A., Abdel-Bar H.M., Elimam H., Mostafa A., Moatasim Y., Abouzid K.A.M., Elshaier Y.A.M.M. (2021). Structure- and ligand-based in silico studies towards the repurposing of marine bioactive compounds to target sars-cov-2. Arab. J. Chem..

[8] Xu R., Fazio G.C., Matsuda S.P.T. (2004). On the origins of triterpenoid skeletal diversity. Phytochemistry.

[9] Bruneton J. (1999). Pharmacognosie, Phytochimie, Plantes Médicinales.

[10] Muffler K., Leipold D., Scheller M.-C., Haas C., Steingroewer J., Bley T., Neuhaus H.E., Mirata M.A., Schrader J., Ulber R. (2011). Biotransformation of triterpenes. Process Biochem..

[11] Hisham Shady N., Youssif K.A., Sayed A.M., Belbahri L., Oszako T., Hassan H.M., Abdelmohsen U.R. (2021). Sterols and triterpenes: Antiviral potential supported by in-silico analysis. Plants.

[12] Dzubak P., Hajduch M., Vydra D., Hustova A., Kvasnica M., Biedermann D., Markova L., Urban M., Sarek J. (2006). Pharmacological activities of natural triterpenoids and their therapeutic implications. Nat. Prod. Rep..

[13] Sparg S.G., Light M.E., van Staden J. (2004). Biological activities and distribution of plant saponins. J. Ethnopharmacol..

[14] Cheng K., Liu J., Liu X., Li H., Sun H., Xie J. (2009). Synthesis of glucoconjugates of oleanolic acid as inhibitors of glycogen phosphorylase. Carbohydr. Res..

[15] Chouaïb K., Hichri F., Nguir A., Daami-Remadi M., Elie N., Touboul D., Ben Jannet H., Hamza M.A. (2015). Semi-synthesis of new antimicrobial esters from the natural oleanolic and maslinic acids. Food Chem..

[16] Chouaïb K., Delemasure S., Dutartre P., Jannet H.B. (2016). Microwave-assisted synthesis, anti-inflammatory and anti-proliferative activities of new maslinic acid derivatives bearing 1,5- and 1,4-disubstituted triazoles. J. Enzym. Inhib. Med. Chem..

[17] Chouaïb K., Romdhane A., Delemasure S., Dutartre P., Elie N., Touboul D., Ben jannet H., Ali Hamza M.h. (2016). Regiospecific synthesis, anti-inflammatory and anticancer evaluation of novel 3,5-disubstituted isoxazoles from the natural maslinic and oleanolic acids. Ind. Crop. Prod..

[18] Chouaïb K., Romdhane A., Delemasure S., Dutartre P., Elie N., Touboul D., Ben Jannet H. (2019). Regiospecific synthesis by copper- and ruthenium-catalyzed azide–alkyne 1,3-dipolar cycloaddition, anticancer and anti-inflammatory activities of oleanolic acid triazole derivatives. Arab. J. Chem..

[19] Chen P., Zeng H., Wang Y., Fan X., Xu C., Deng R., Zhou X., Bi H., Huang M. (2014). Low dose of oleanolic acid protects against lithocholic acid-induced cholestasis in mice: Potential involvement of nuclear factor-e2-related factor 2-mediated upregulation of multidrug resistance-associated proteins. Drug Metab. Dispos. Biol. Fate Chem..

[20] Sheng H., Sun H. (2011). Synthesis, biology and clinical significance of pentacyclic triterpenes: A multi-target approach to prevention and treatment of metabolic and vascular diseases. Nat. Prod. Rep..

[21] Chen D.-F., Zhang S.-X., Wang H.-K., Zhang S.-Y., Sun Q.-Z., Cosentino L.M., Lee K.-H. (1999). Novel anti-hiv lancilactone c and related triterpenes from kadsura lancilimba. J. Nat. Prod..

[22] Kashiwada Y., Wang H.K., Nagao T., Kitanaka S., Yasuda I., Fujioka T., Yamagishi T., Cosentino L.M., Kozuka M., Okabe H. (1998). Anti-aids agents. 30. Anti-hiv activity of oleanolic acid, pomolic acid, and structurally related triterpenoids. J. Nat. Prod..

[23] Zhu Y.M., Shen J.K., Wang H.K., Cosentino L.M., Lee K.H. (2001). Synthesis and anti-hiv activity of oleanolic acid derivatives. Bioorg. Med. Chem. Lett..

[24] Shanmugam M.K., Dai X., Kumar A.P., Tan B.K., Sethi G., Bishayee A. (2014). Oleanolic acid and its synthetic derivatives for the prevention and therapy of cancer: Preclinical and clinical evidence. Cancer Lett..

[25] Hichri F., Jannet H.B., Cheriaa J., Jegham S., Mighri Z. (2003). Antibacterial activities of a few prepared derivatives of oleanolic acid and of other natural triterpenic compounds. Comptes Rendus Chim..

[26] Tsuji M., Sriwilaijaroen N., Inoue H., Miki K., Kinoshita K., Koyama K., Furuhata K., Suzuki Y., Takahashi K. (2018). Synthesis and anti-influenza virus evaluation of triterpene-sialic acid conjugates. Bioorg. Med. Chem..

[27] Blanco-Cabra N., Vega-Granados K., Moya-Andérico L., Vukomanovic M., Parra A., Álvarez de Cienfuegos L., Torrents E. (2019). Novel oleanolic and maslinic acid derivatives as a promising treatment against bacterial biofilm in nosocomial infections: An in vitro and in vivo study. ACS Infect. Dis..

[28] Wen X., Zhang P., Liu J., Zhang L., Wu X., Ni P., Sun H. (2006). Pentacyclic triterpenes. Part 2: Synthesis and biological evaluation of maslinic acid derivatives as glycogen phosphorylase inhibitors. Bioorg. Med. Chem. Lett..

[29] Qiu W.-W., Shen Q., Yang F., Wang B., Zou H., Li J.-Y., Li J., Tang J. (2009). Synthesis and biological evaluation of heterocyclic ring-substituted maslinic acid derivatives as novel inhibitors of protein tyrosine phosphatase 1b. Bioorg. Med. Chem. Lett..

[30] Taniguchi S., Imayoshi Y., Kobayashi E., Takamatsu Y., Ito H., Hatano T., Sakagami H., Tokuda H., Nishino H., Sugita D. (2002). Production of bioactive triterpenes by eriobotrya japonica calli. Phytochemistry.

[31] Parra A., Rivas F., Lopez P.E., Garcia-Granados A., Martinez A., Albericio F., Marquez N., Muñoz E. (2009). Solution- and solid-phase synthesis and anti-hiv activity of maslinic acid derivatives containing amino acids and peptides. Bioorg. Med. Chem..

[32] Agrawal N., Mishra P. (2018). The synthetic and therapeutic expedition of isoxazole and its analogs. Med. Chem. Res..

[33] Kónya B., Docsa T., Gergely P., Somsák L. (2012). Synthesis of heterocyclic n-(β-d-glucopyranosyl)carboxamides for inhibition of glycogen phosphorylase. Carbohydr. Res..

[34] Koufaki M., Tsatsaroni A., Alexi X., Guerrand H., Zerva S., Alexis M.N. (2011). Isoxazole substituted chromans against oxidative stress-induced neuronal damage. Bioorg. Med. Chem..

[35] Filali I., Romdhane A., Znati M., Jannet H.B., Bouajila J. (2016). Synthesis of new harmine isoxazoles and evaluation of their potential anti-alzheimer, anti-inflammatory, and anticancer activities. Med. Chem..

[36] Agalave S.G., Maujan S.R., Pore V.S. (2011). Click chemistry: 1,2,3-triazoles as pharmacophores. Chem. Asian J..

[37] Gonzaga D.T.G., Souza T.M.L., Andrade V.M.M., Ferreira V.F., de C da Silva F. (2018). Identification of 1-aryl-1h-1,2,3-triazoles as potential new antiretroviral agents. Med. Chem..

[38] El-Sebaey S.A. (2020). Recent advances in 1,2,4-triazole scaffolds as antiviral agents. ChemistrySelect.

[39] Wang X.L., Wan K., Zhou C.H. (2010). Synthesis of novel sulfanilamide-derived 1,2,3-triazoles and their evaluation for antibacterial and antifungal activities. Eur. J. Med. Chem..

[40] Kandeil A., Mostafa A., El-Shesheny R., Shehata M., Roshdy W.H., Ahmed S.S., Gomaa M., Taweel A.E., Kayed A.E., Mahmoud S.H. (2020). Coding-complete genome sequences of two sars-cov-2 isolates from egypt. Microbiol. Resour. Announc..

[41] Guinda A., Rada M., Delgado T., Gutiérrez-Adánez P., Castellano J.M. (2010). Pentacyclic triterpenoids from olive fruit and leaf. J. Agric. Food Chem..

[42] Gil M., Haïdour A., Ramos J.L. (1997). Identification of two triterpenoids in solid wastes from olive cake. J. Agric. Food Chem..

[43] Romero C., García A., Medina E., Ruíz-Méndez M.V., Castro A.d., Brenes M. (2010). Triterpenic acids in table olives. Food Chem..

[44] Goulas V., Manganaris G.A. (2012). Towards an efficient protocol for the determination of triterpenic acids in olive fruit: A comparative study of drying and extraction methods. Phytochem. Anal..

[45] Chan R.W.Y., Hemida M.G., Kayali G., Chu D.K.W., Poon L.L.M., Alnaeem A., Ali M.A., Tao K.P., Ng H.Y., Chan M.C.W. (2014). Tropism and replication of middle east respiratory syndrome coronavirus from dromedary camels in the human respiratory tract: An in-vitro and ex-vivo study. Lancet Respir. Med..

[46] Oakdale J.S., Fokin V.V., Umezaki S., Fukuyama T. (2013). Preparation of 1,5-disubstituted 1,2,3-triazoles via ruthenium-catalyzed azide alkyne cycloaddition. Org. Synth.

[47] Takasu K., Azuma T., Takemoto Y. (2010). Synthesis of trifunctional thioureas bearing 1,5-disubstituted triazole tether by ru-catalyzed huisgen cycloaddition. Tetrahedron Lett..

[48] Nador F., Volpe M.A., Alonso F., Feldhoff A., Kirschning A., Radivoy G. (2013). Copper nanoparticles supported on silica coated maghemite as versatile, magnetically recoverable and reusable catalyst for alkyne coupling and cycloaddition reactions. Appl. Catal. A Gen..

[49] Hansen T.V., Wu P., Fokin V.V. (2005). One-pot copper(i)-catalyzed synthesis of 3,5-disubstituted isoxazoles. J. Org. Chem..

[50] Kuo Y.C., Lin L.C., Tsai W.J., Chou C.J., Kung S.H., Ho Y.H. (2002). Samarangenin b from limonium sinense suppresses herpes simplex virus type 1 replication in vero cells by regulation of viral macromolecular synthesis. Antimicrob. Agents Chemother..

[51] Vilar S., Cozza G., Moro S. (2008). Medicinal chemistry and the molecular operating environment (moe): Application of qsar and molecular docking to drug discovery. Curr. Top. Med. Chem..

[52] Al-Karmalawy A.A., Khattab M. (2020). Molecular modelling of mebendazole polymorphs as a potential colchicine binding site inhibitor. New J. Chem..

[53] Jin Z., Du X., Xu Y., Deng Y., Liu M., Zhao Y., Zhang B., Li X., Zhang L., Peng C. (2020). Structure of mpro from sars-cov-2 and discovery of its inhibitors. Nature.

[54] Ghanem A., Emara H.A., Muawia S., Abd El Maksoud A.I., Al-Karmalawy A.A., Elshal M.F. (2020). Tanshinone iia synergistically enhances the antitumor activity of doxorubicin by interfering with the pi3k/akt/mtor pathway and inhibition of topoisomerase ii: In vitro and molecular docking studies. New J. Chem..

[55] Khattab M., Al-Karmalawy A. (2021). Revisiting activity of some nocodazole analogues as a potential anticancer drugs using molecular docking and dft calculations. Front. Chem..

[56] McConkey B.J., Sobolev V., Edelman M. (2002). The performance of current methods in ligand–protein docking. Curr. Sci..

[57] Eliaa S.G., Al-Karmalawy A.A., Saleh R.M., Elshal M.F. (2020). Empagliflozin and doxorubicin synergistically inhibit the survival of triple-negative breast cancer cells via interfering with the mtor pathway and inhibition of calmodulin: In vitro and molecular docking studies. ACS Pharmacol. Transl. Sci..

[58] Al-Rabia M.W., Alhakamy N.A., Ahmed O.A.A., Eljaaly K., Aloafi A.L., Mostafa A., Asfour H.Z., Aldarmahi A.A., Darwish K.M., Ibrahim T.S. (2021). Repurposing of sitagliptin- melittin optimized nanoformula against sars-cov-2; antiviral screening and molecular docking studies. Pharmaceutics.

[59] Feoktistova M., Geserick P., Leverkus M. (2016). Crystal violet assay for determining viability of cultured cells. Cold Spring Harb. Protoc..

[60] Zhang J., Zhan B., Yao X., Gao Y., Shong J. (1995). Antiviral activity of tannin from the pericarp of punica granatum l. Against genital herpes virus in vitro. China J. Chin. Mater. Med..

[61] Schuhmacher A., Reichling J., Schnitzler P. (2003). Virucidal effect of peppermint oil on the enveloped viruses herpes simplex virus type 1 and type 2 in vitro. Phytomed. Int. J. Phytother. Phytopharm..

