# Supplementary material for: Strong Inhibitory Activity and Action Modes of Synthetic Maslinic Acid Derivative on Highly Pathogenic Coronaviruses: COVID-19 Drug Candidate
Source: Pathogens. 2021 May 19;10(5):623. doi: 10.3390/pathogens10050623 (PMC8159111; doi:10.3390/pathogens10050623)
Supplement: Supplementary file 1 [file pathogens-10-00623-s001.zip › pathogens-1214973-supplementary.pdf]

Supplementary Data

A) NMR Spectra of isolated and synthesized compounds

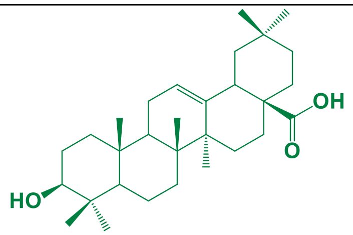

Compound **1**

^{1}H NMR

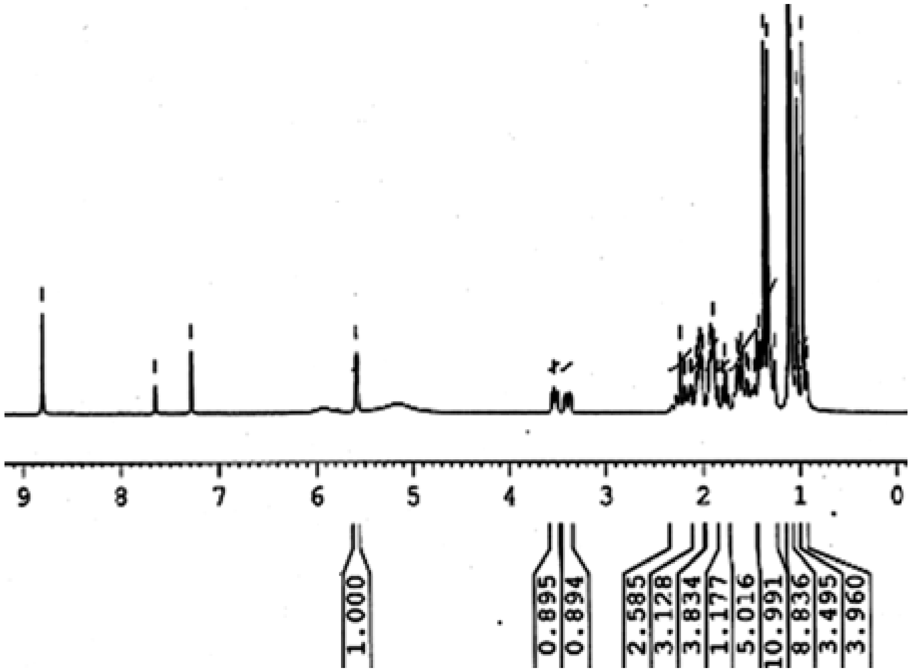

^{13}C NMR

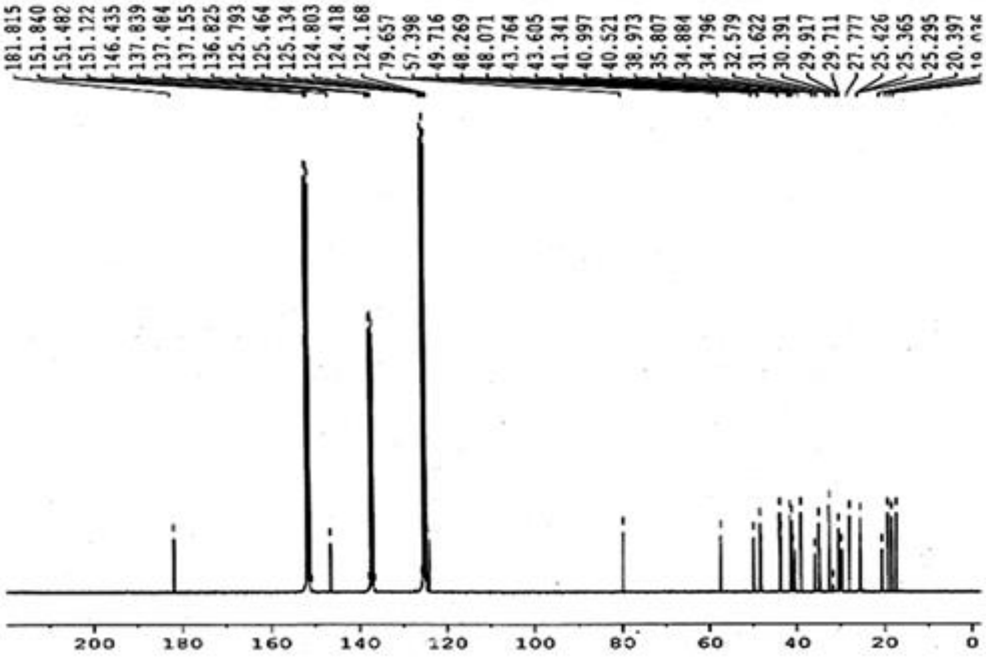

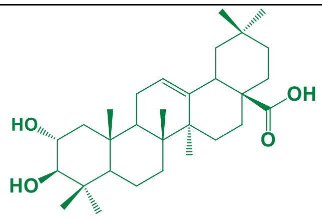

Compound **2**

^{1}H NMR

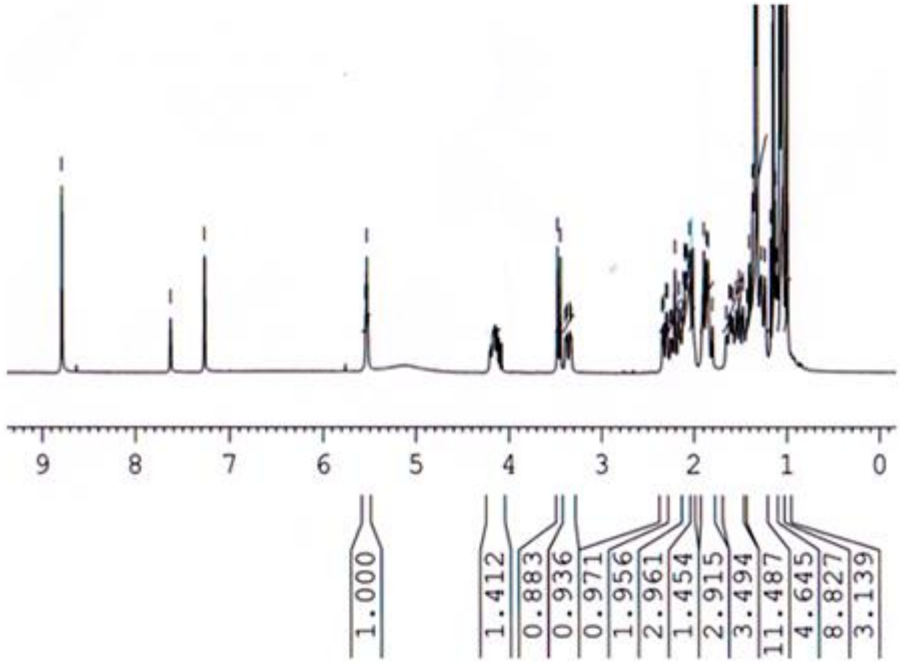

^{13}C NMR

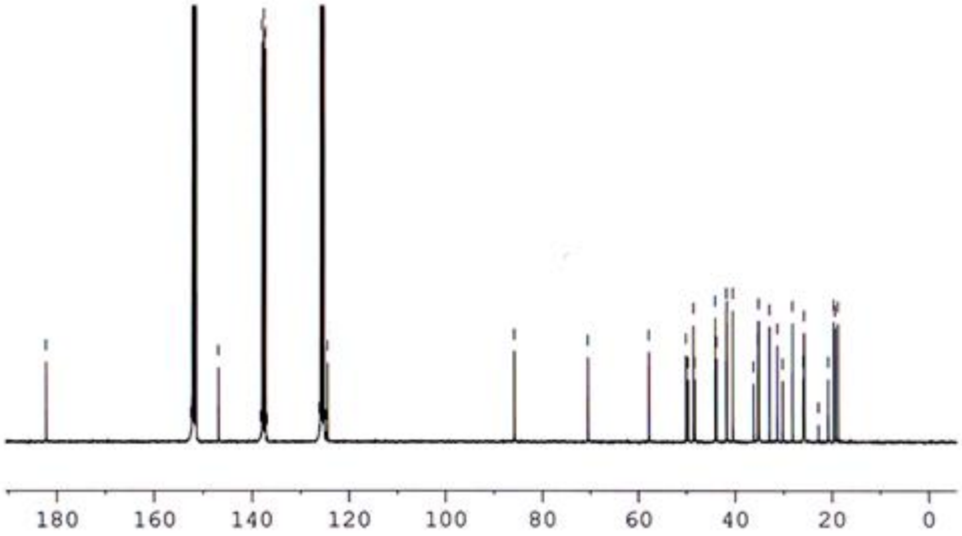

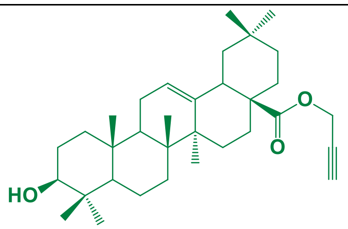

Compound **3**

^{1}H NMR

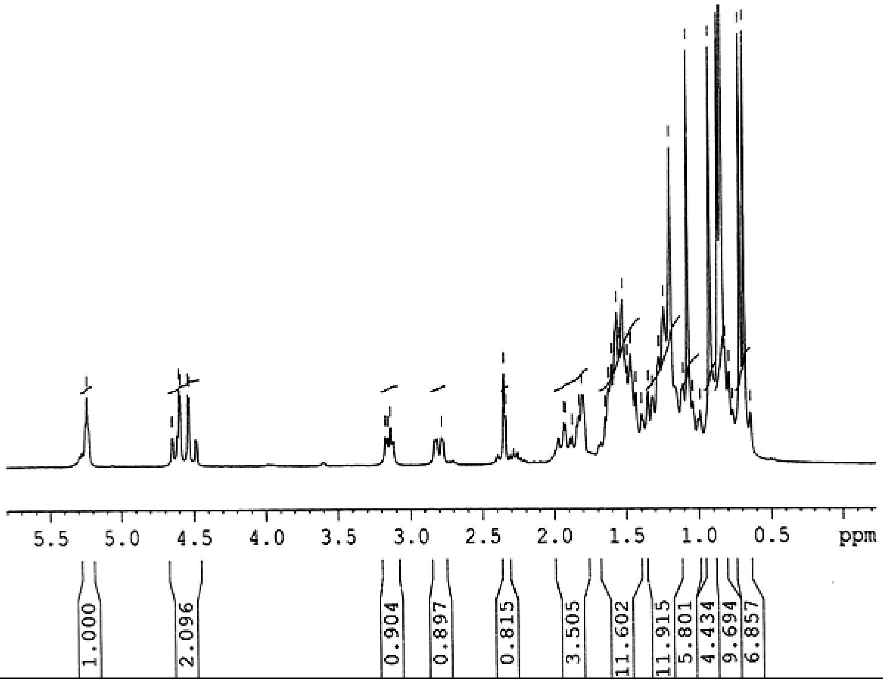

^{13}C NMR

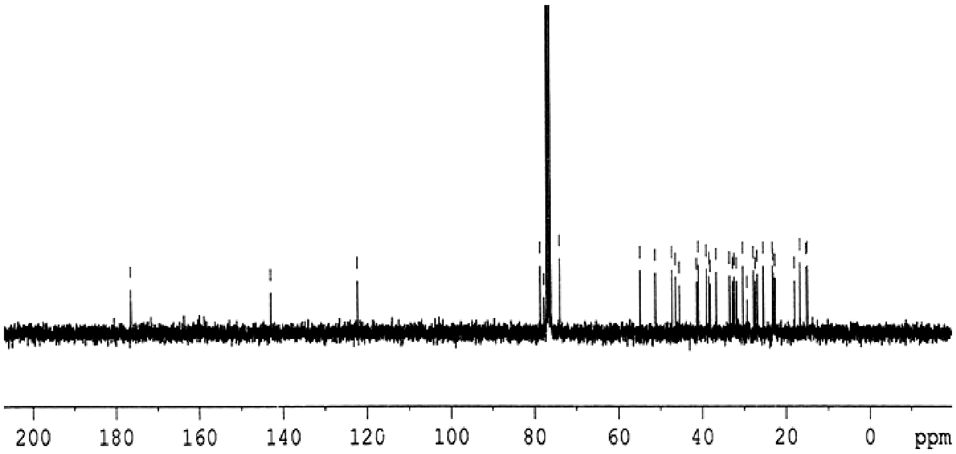

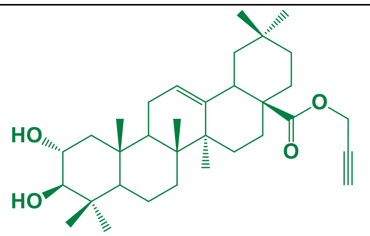

Compound **4**

^{1}H NMR

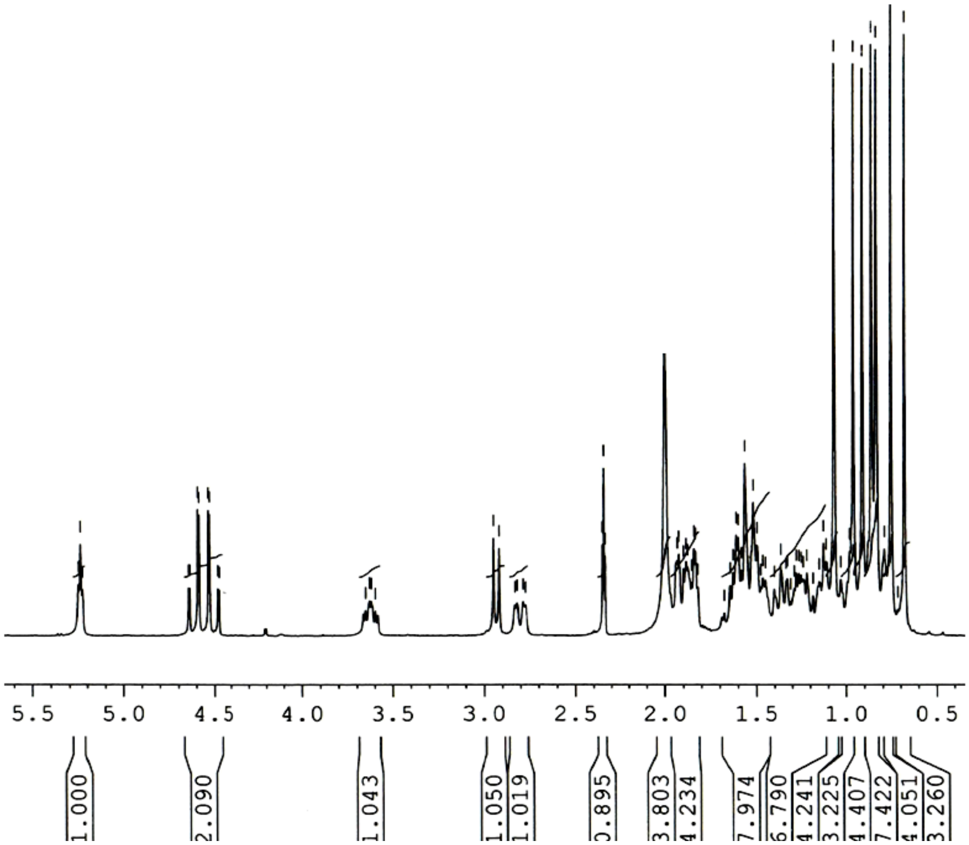

^{13}C NMR

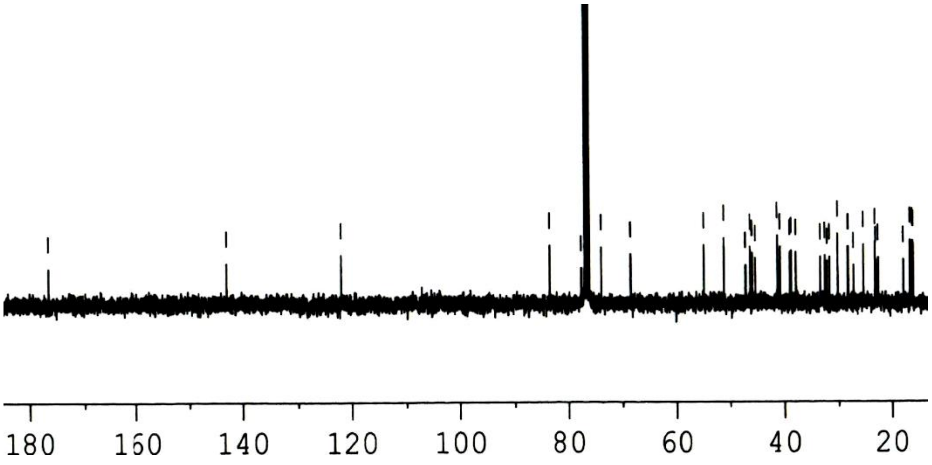

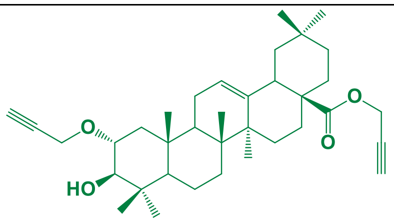

Compound **5**

^{1}H NMR

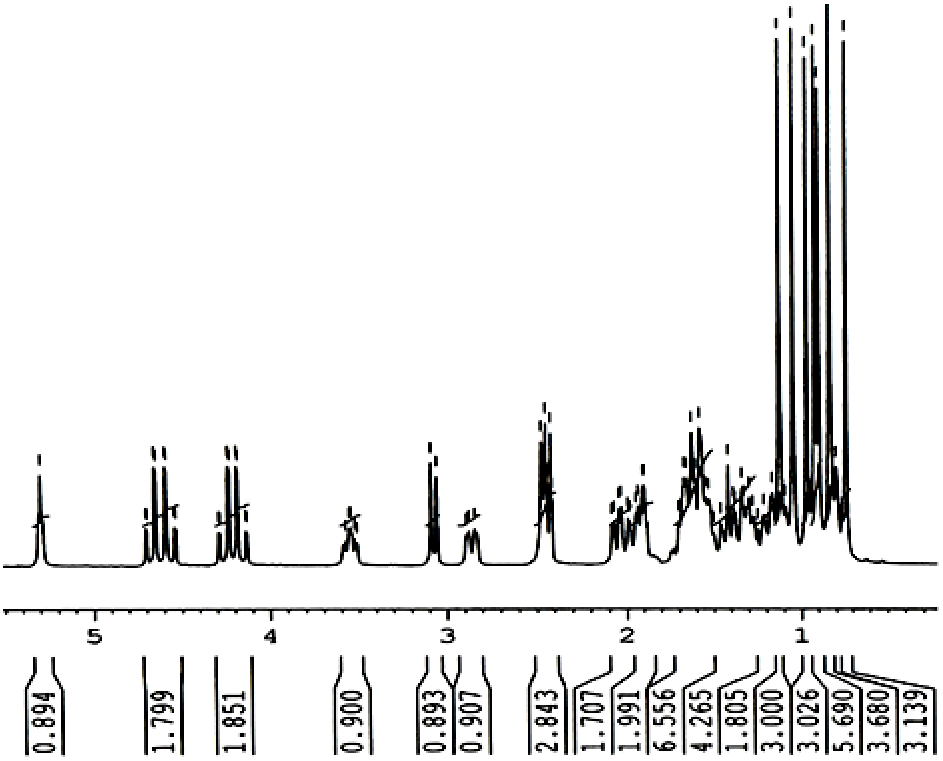

^{13}C NMR

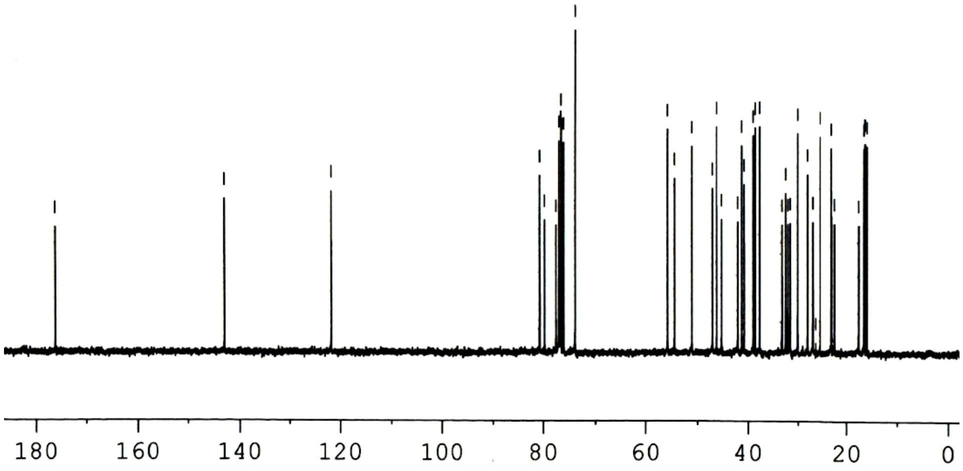

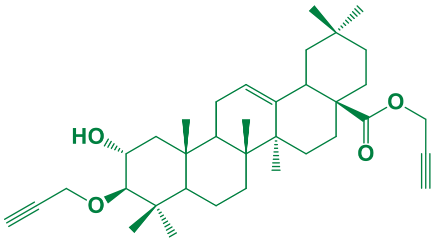

Compound **6**

^{1}H NMR

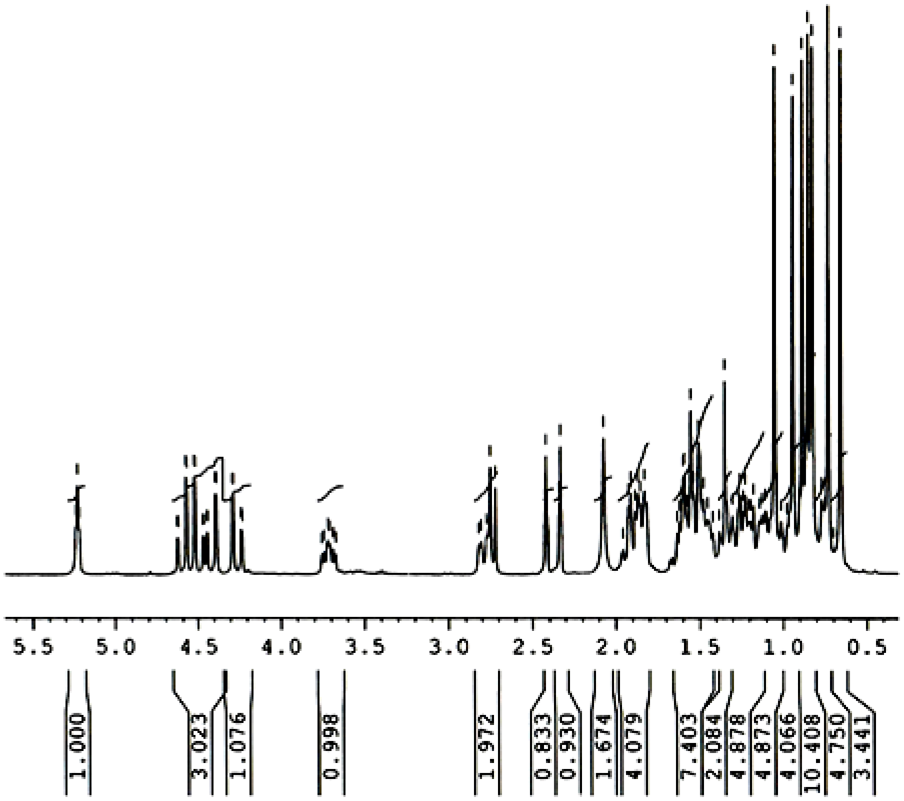

^{13}C NMR

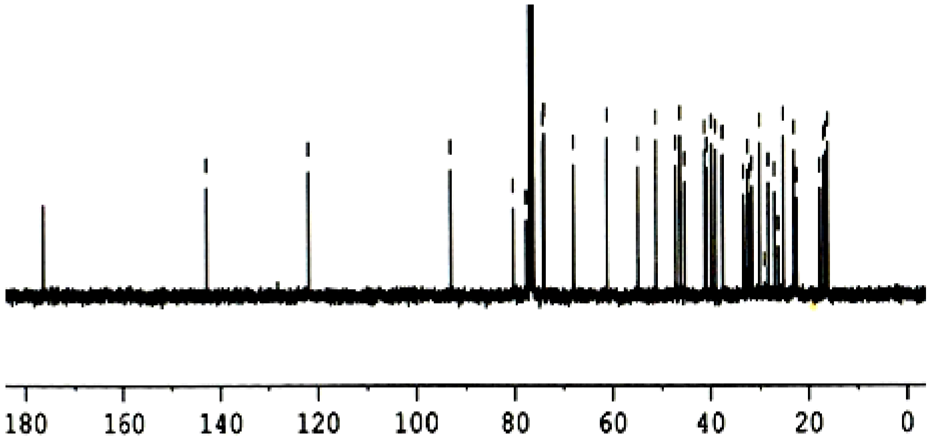

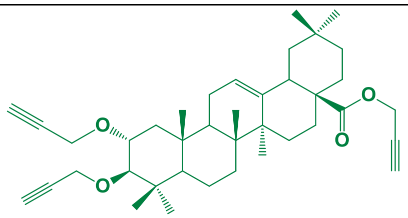

Compound **7**

^{1}H NMR

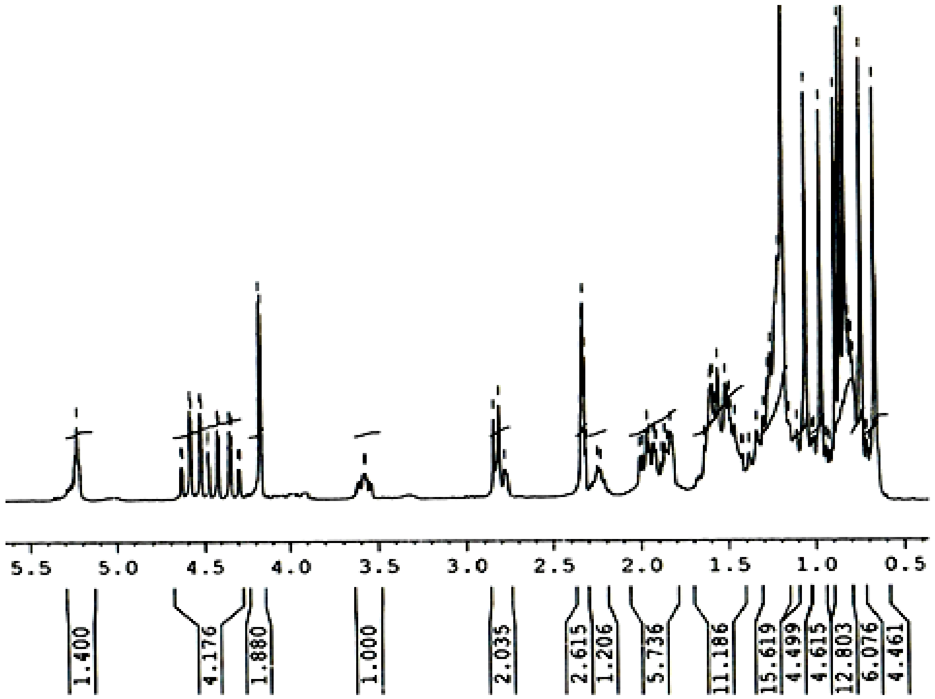

^{13}C NMR

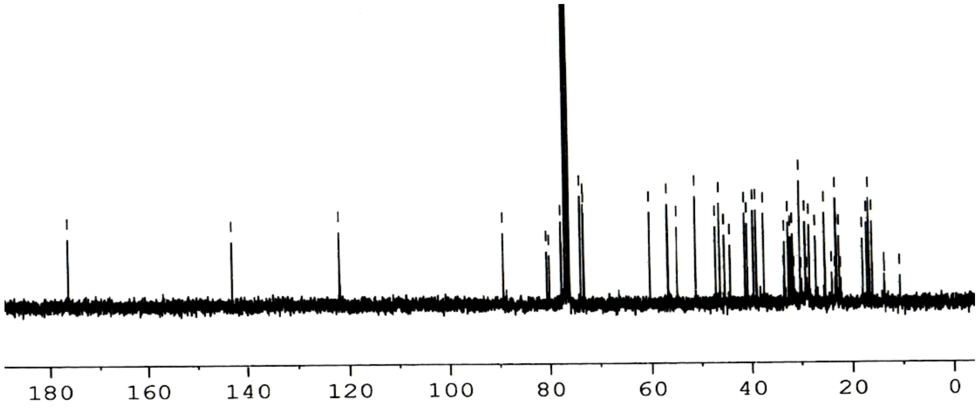

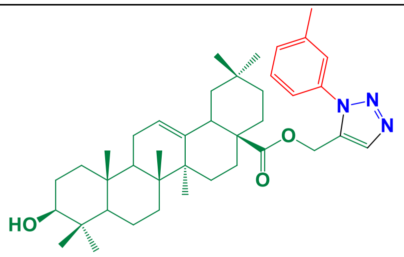

Compound **8**

^{1}H NMR

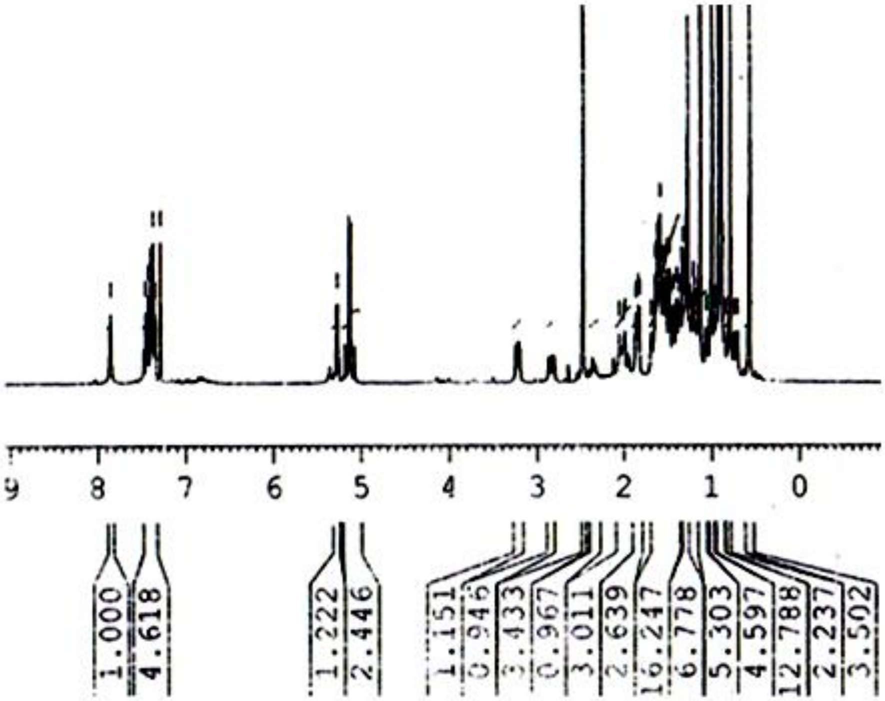

^{13}C NMR

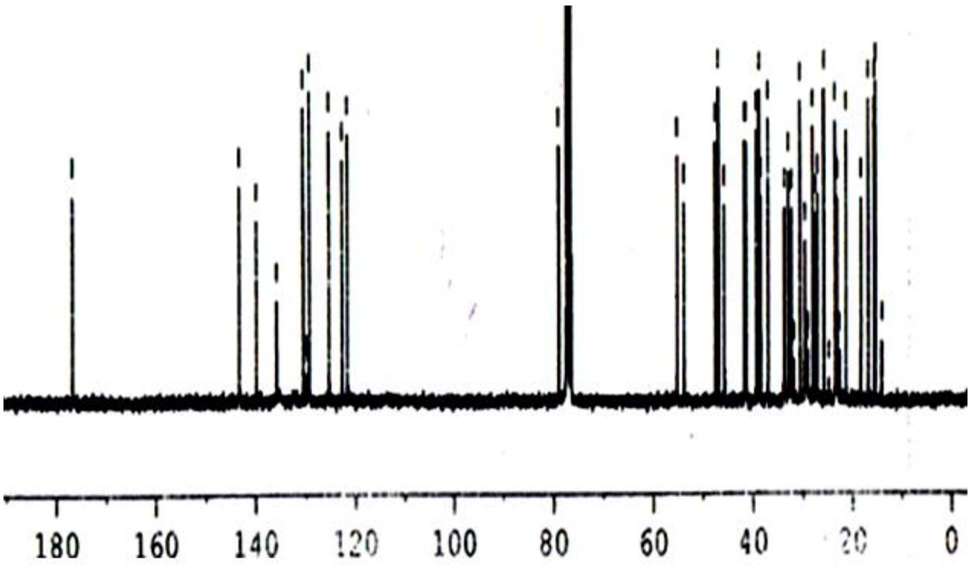

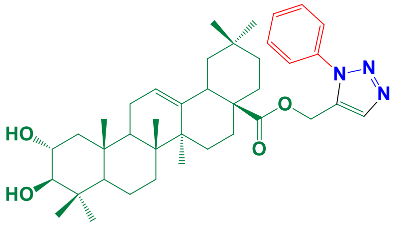

Compound **9**

^{1}H NMR

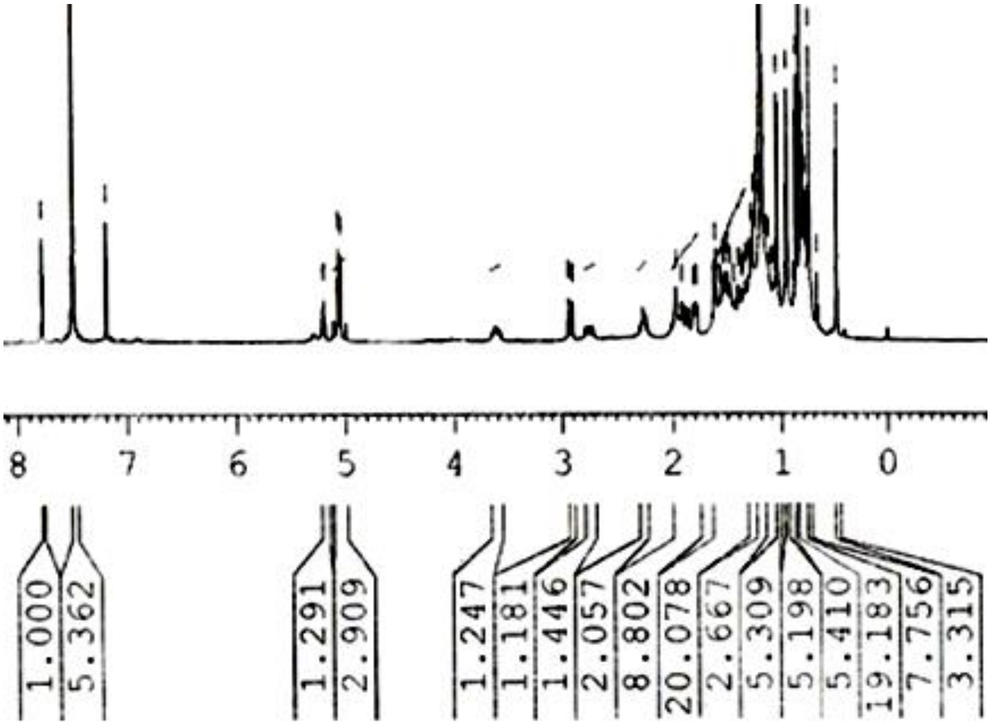

^{13}C NMR

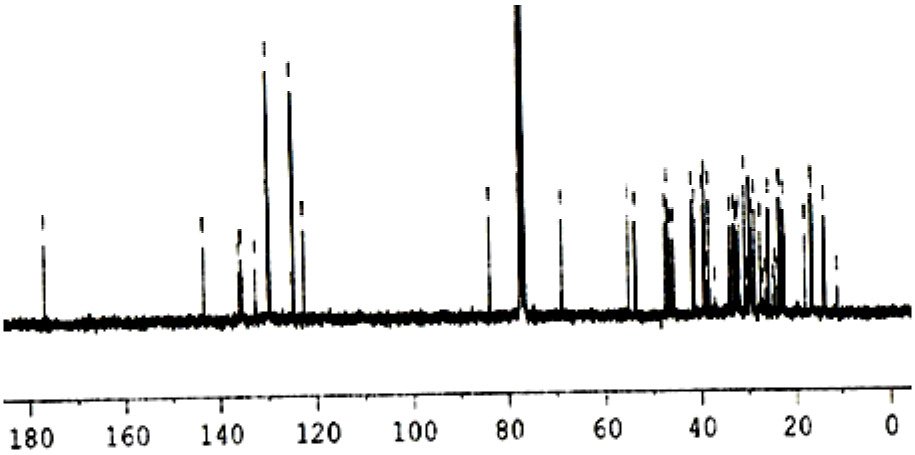

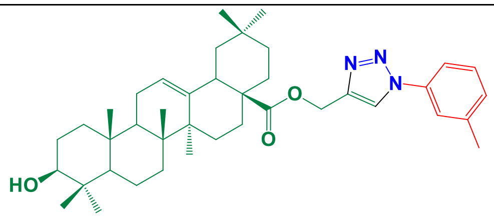

Compound **10**

^{1}H NMR

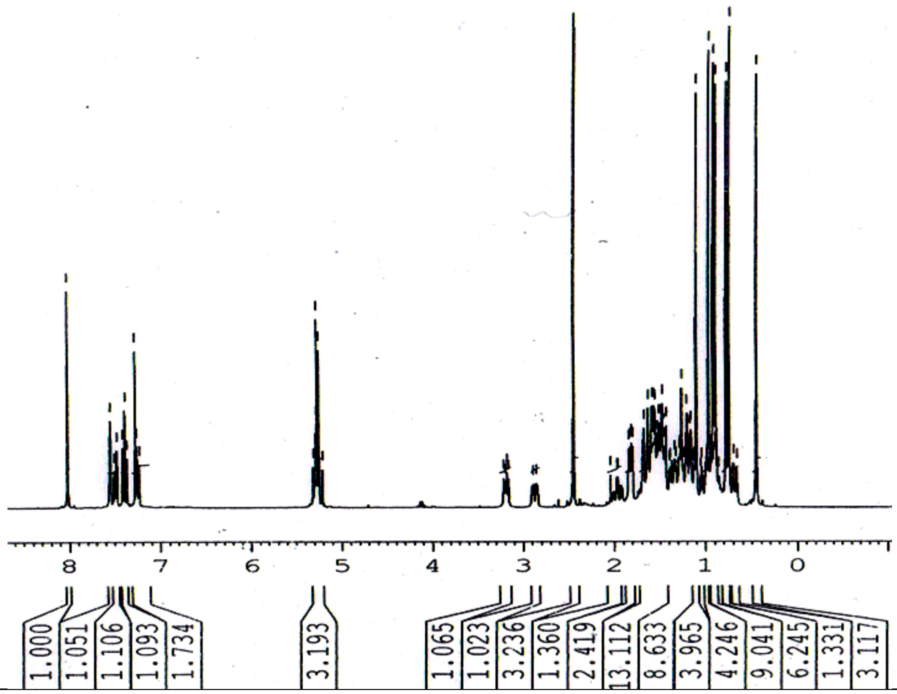

^{13}C NMR

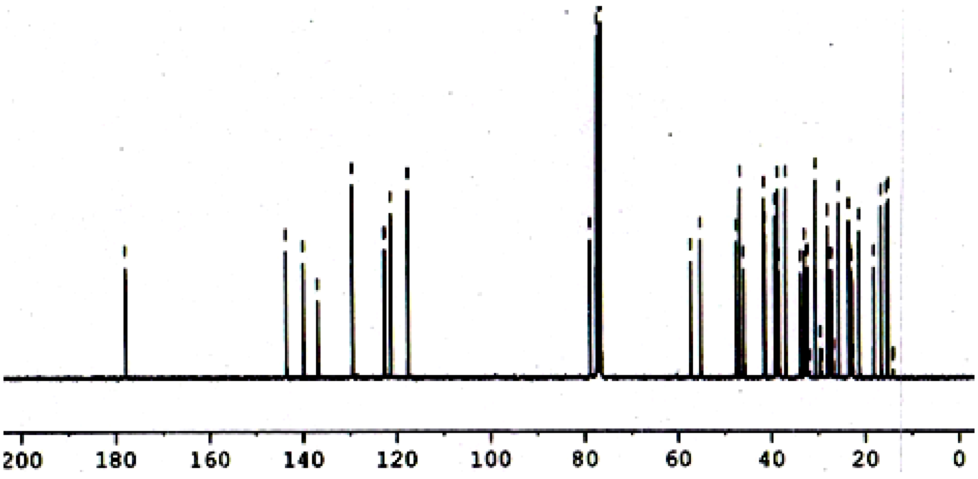

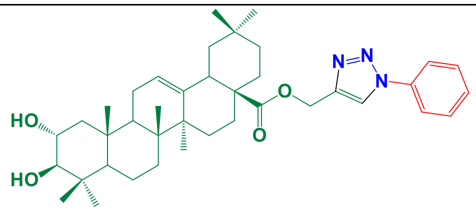

Compound **11**

^{1}H NMR

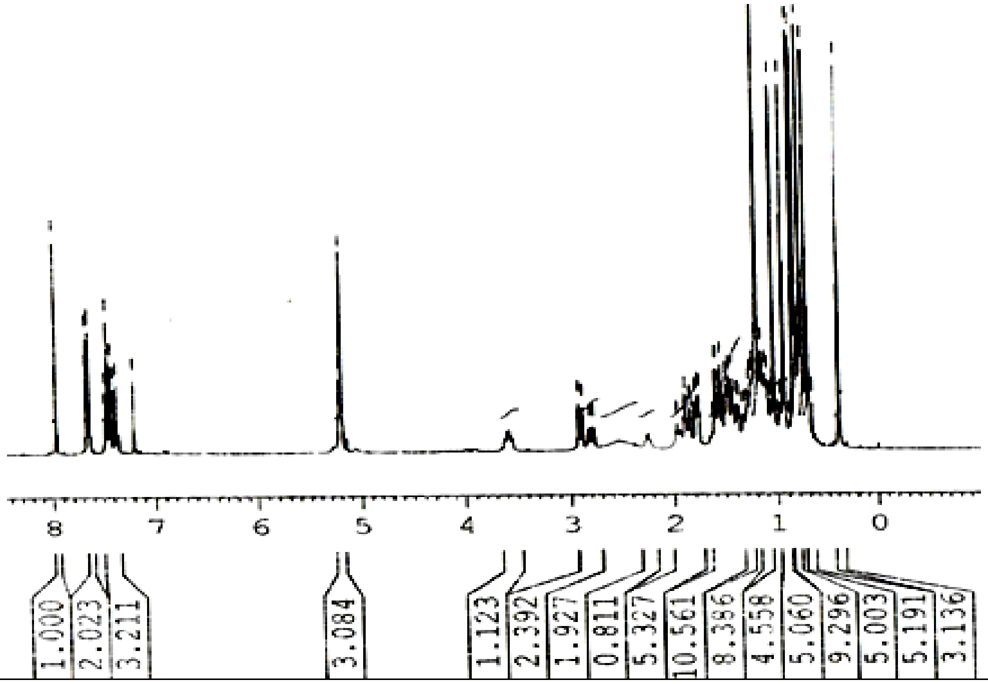

^{13}C NMR

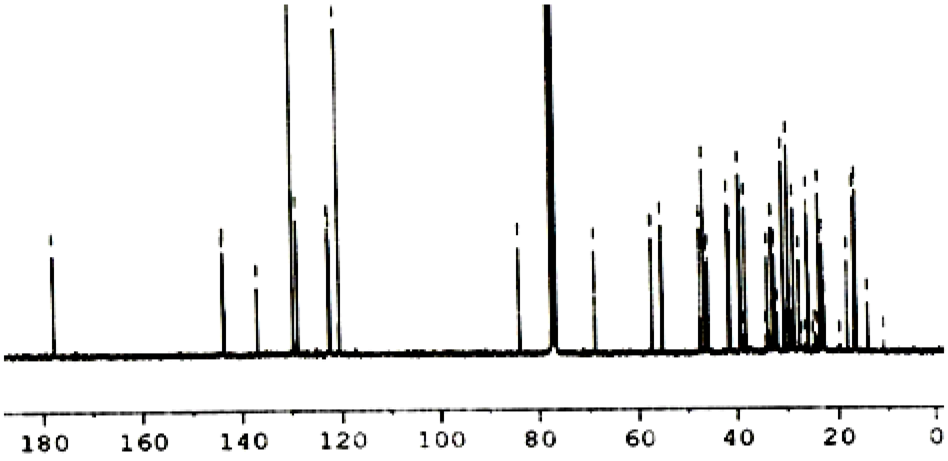

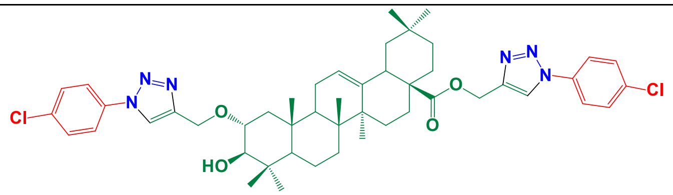

Compound **12**

^{1}H NMR

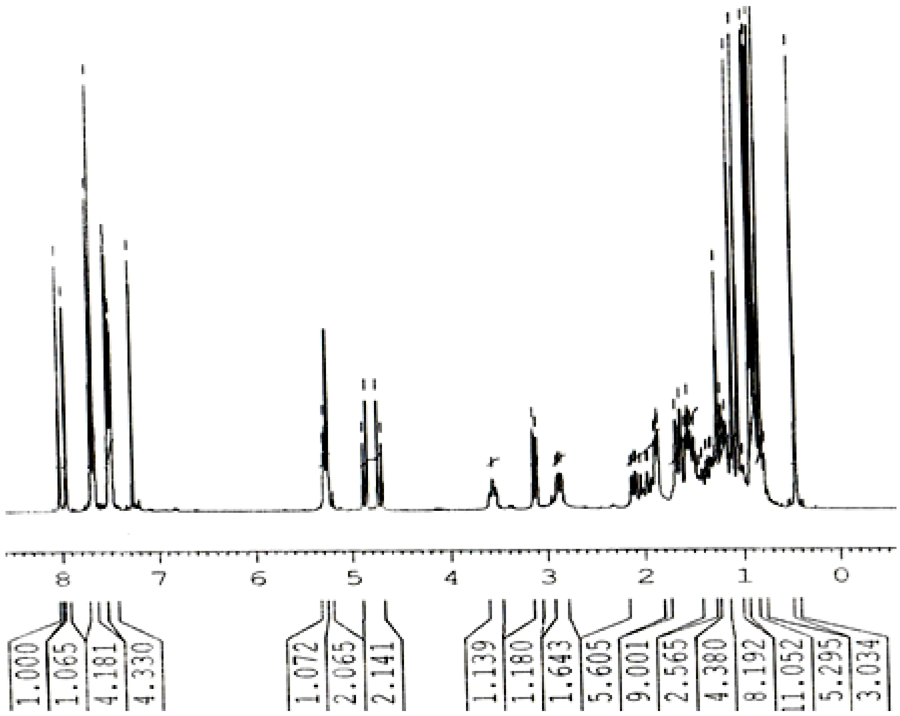

^{13}C NMR

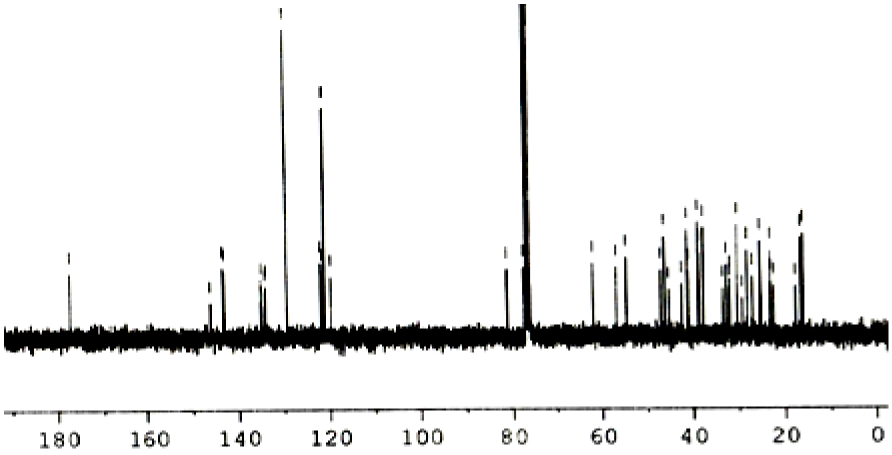

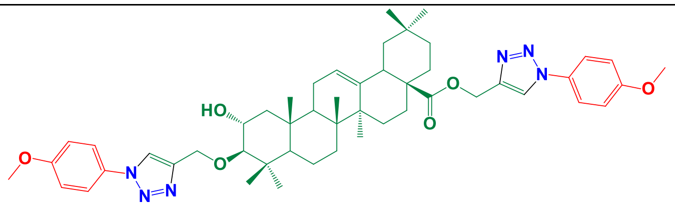

Compound **13**

^{1}H NMR

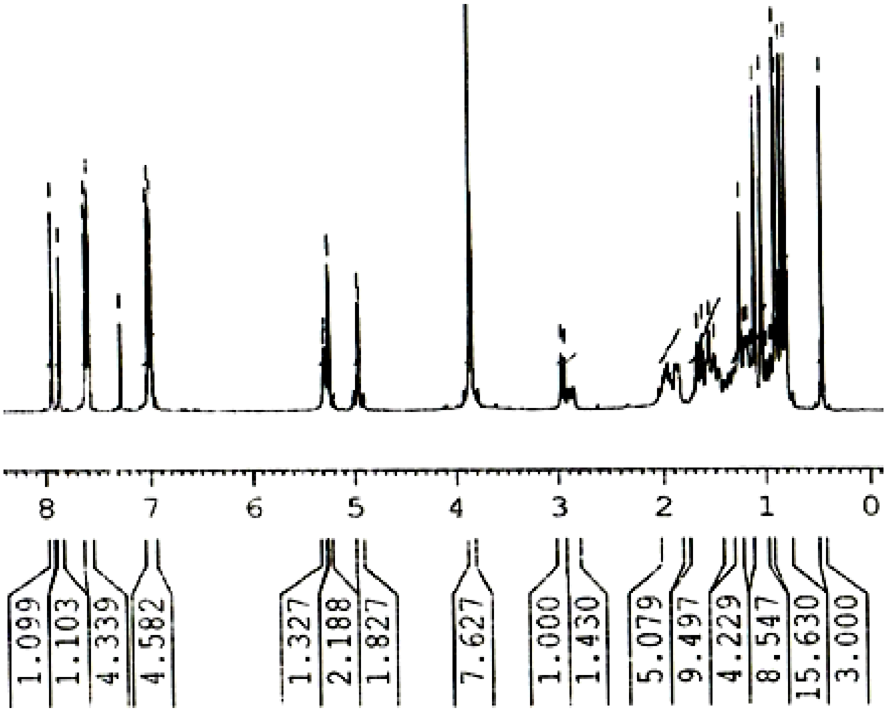

^{13}C NMR

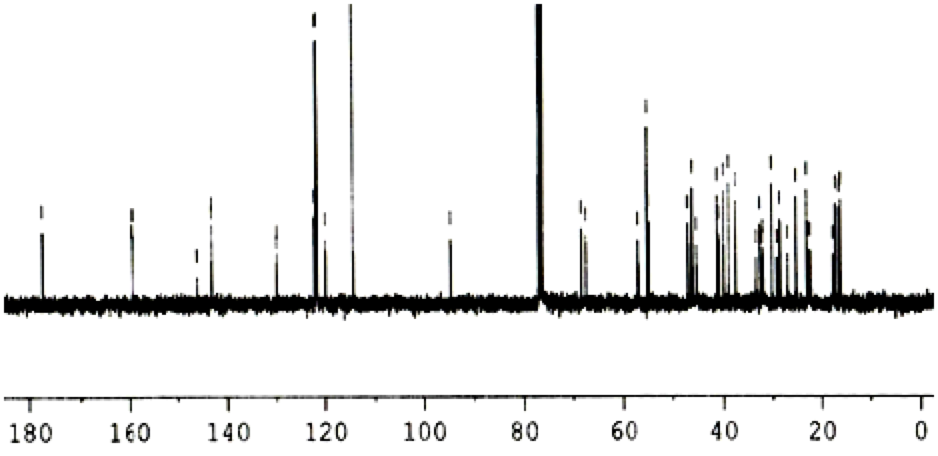

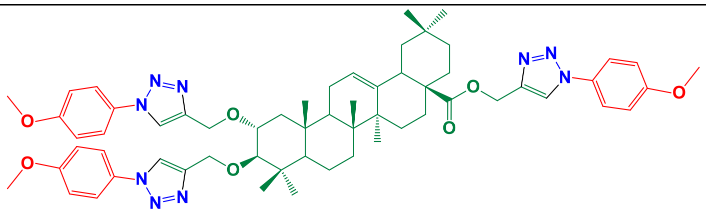

Compound **14**

^{1}H NMR

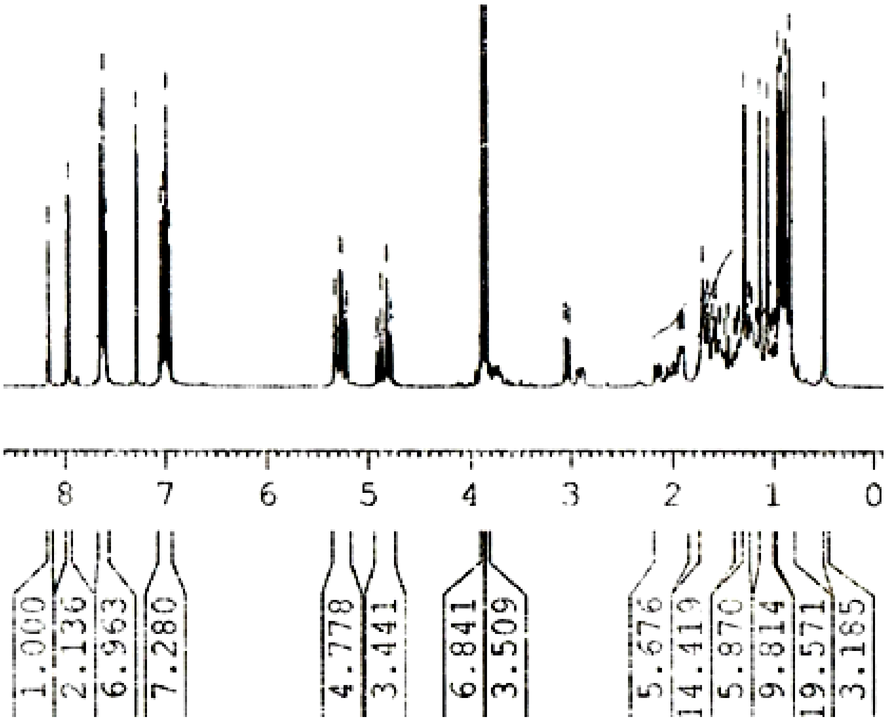

^{13}C NMR

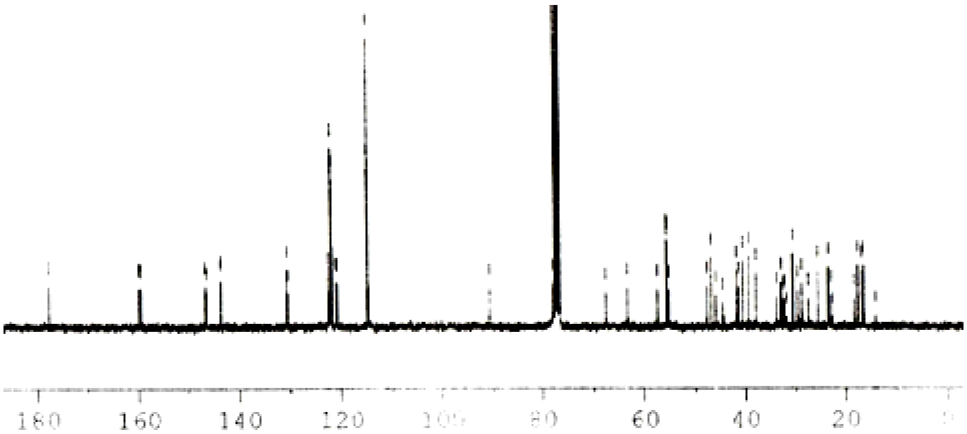

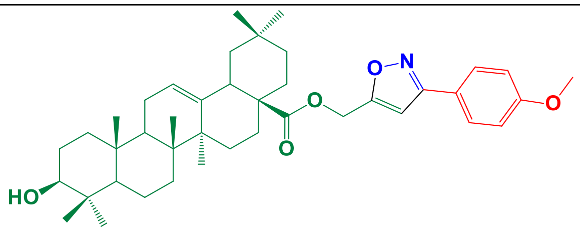

Compound **15**

^{1}H NMR

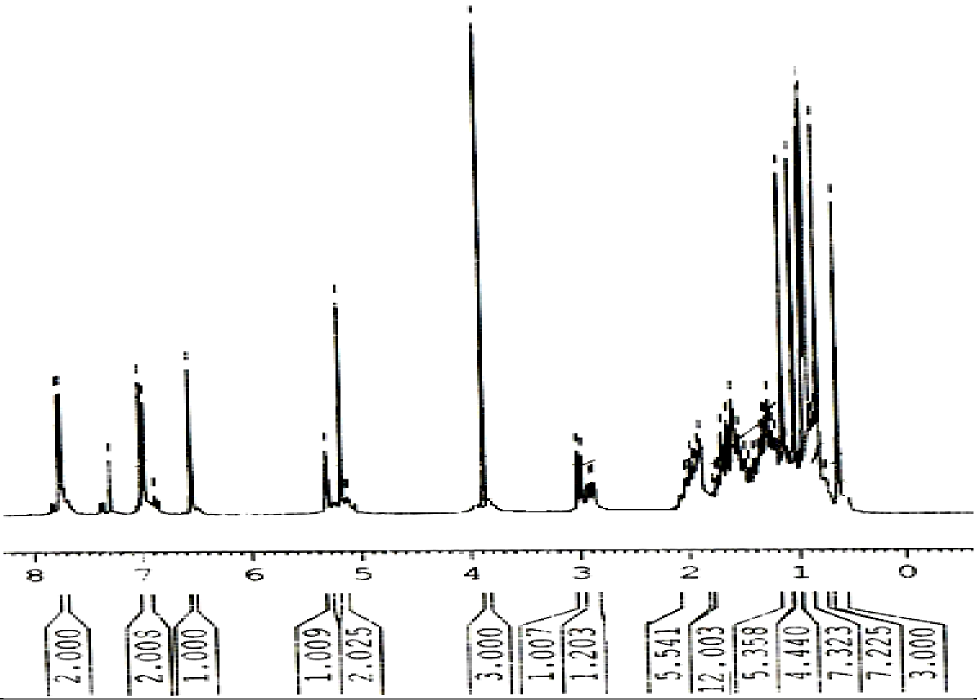

^{13}C NMR

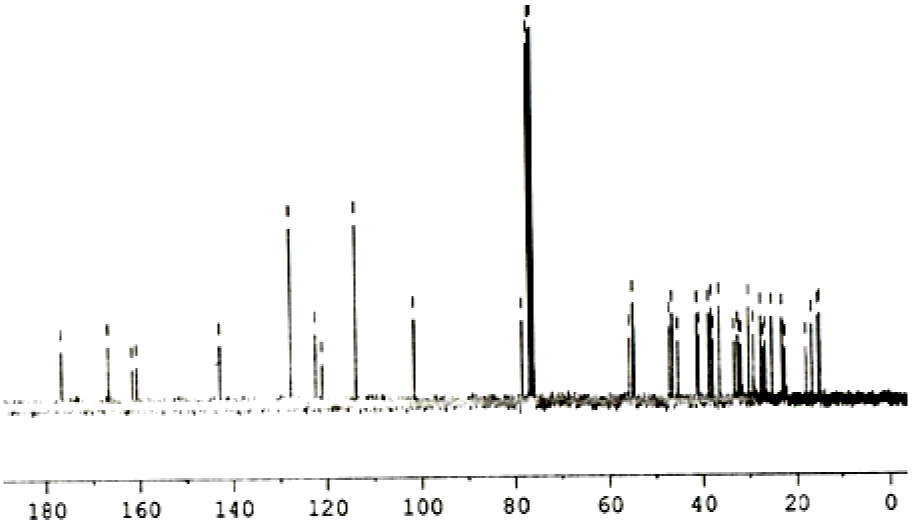

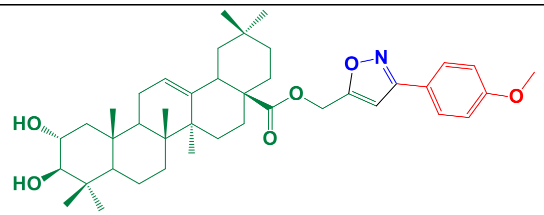

Compound **16**

^{1}H NMR

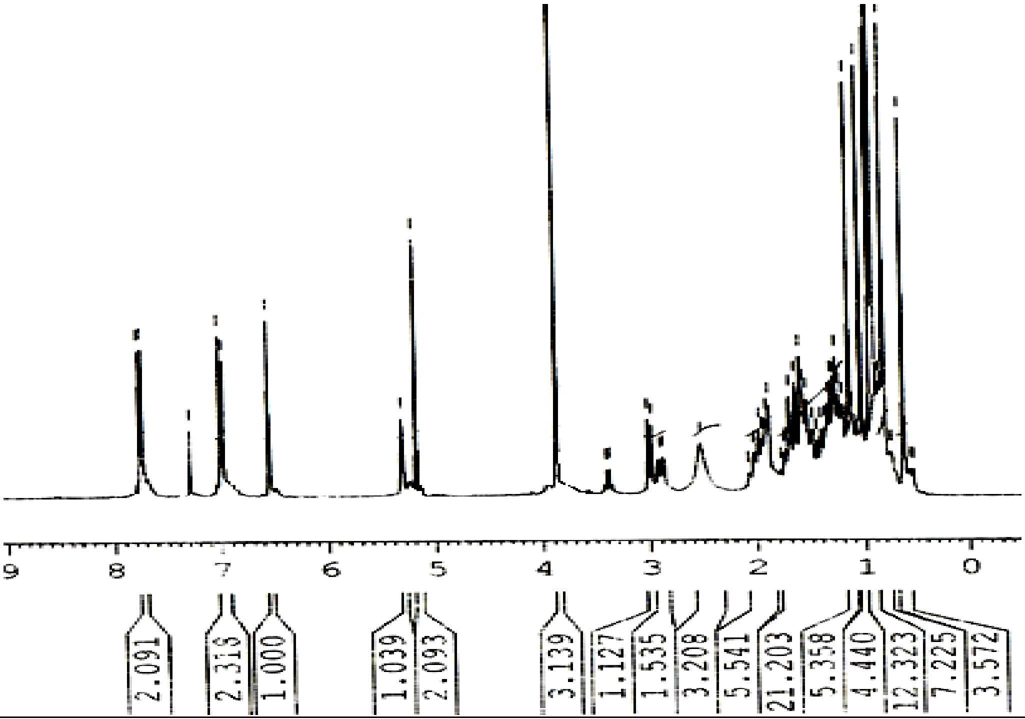

^{13}C NMR

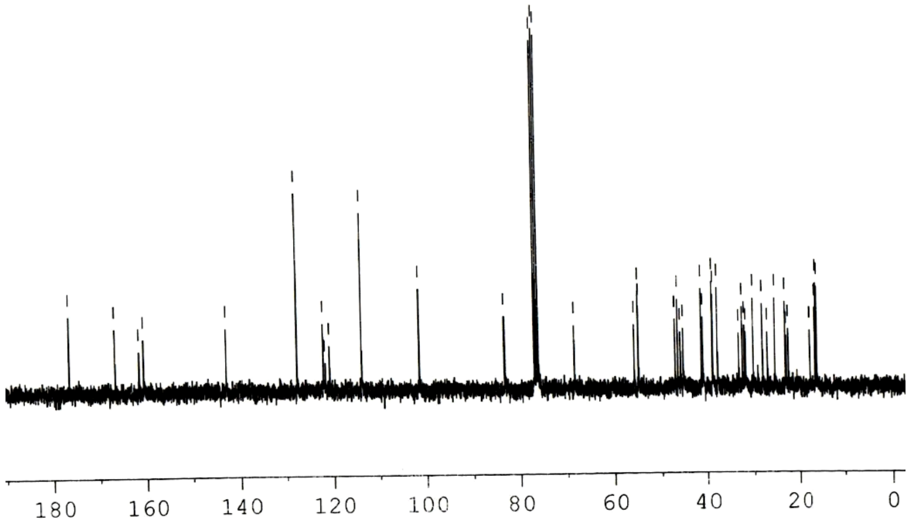

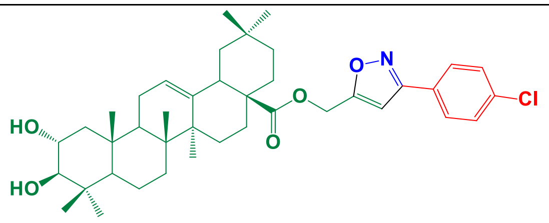

Compound **17**

^{1}H NMR

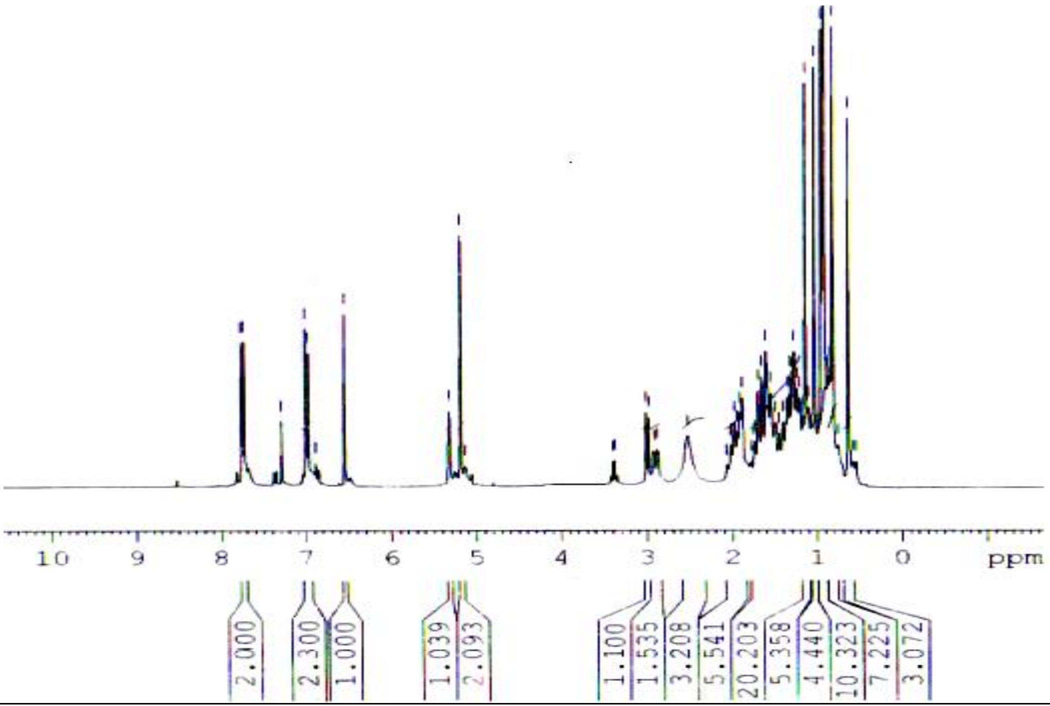

^{13}C NMR

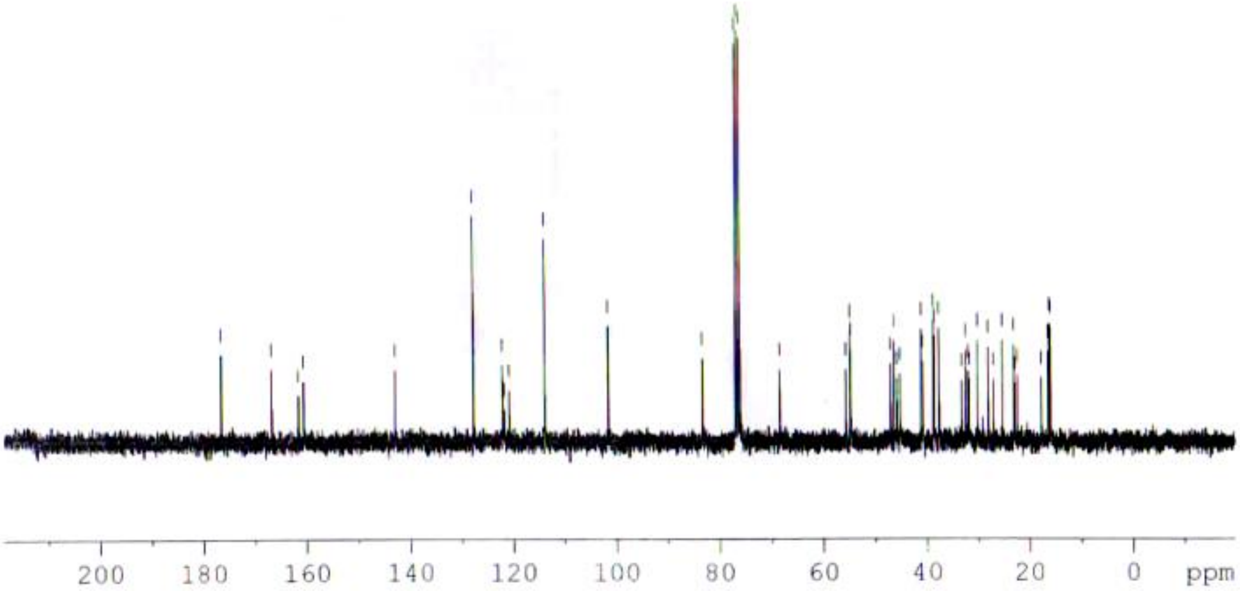

Supplementary Table (S1). 2 D and 3 D representations of the redocking process between the native co-crystallized N3 inhibitor (red) and the docked one (green).

Redocked N3	
**2D**	**3D**
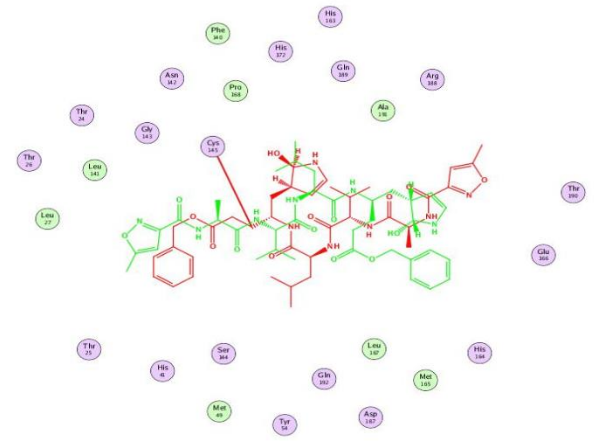	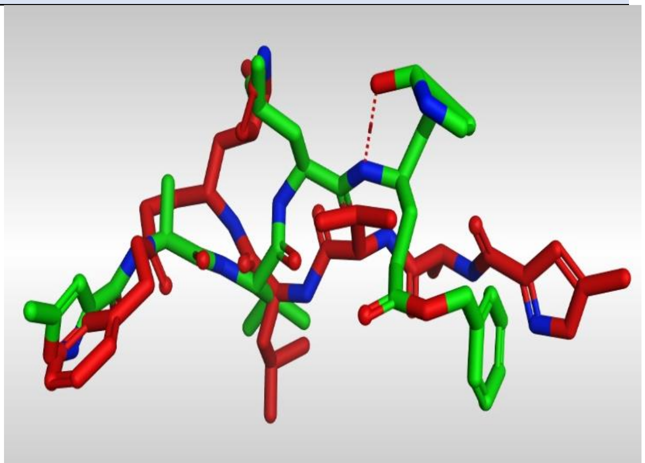

Supplementary Table (S2). 2 D pictures representing the binding interactions between the tested compounds (**1-17**) at the N3-binding pocket in comparison to the docked N3 inhibitor (**18**).

No.	2 D binding interactions
1	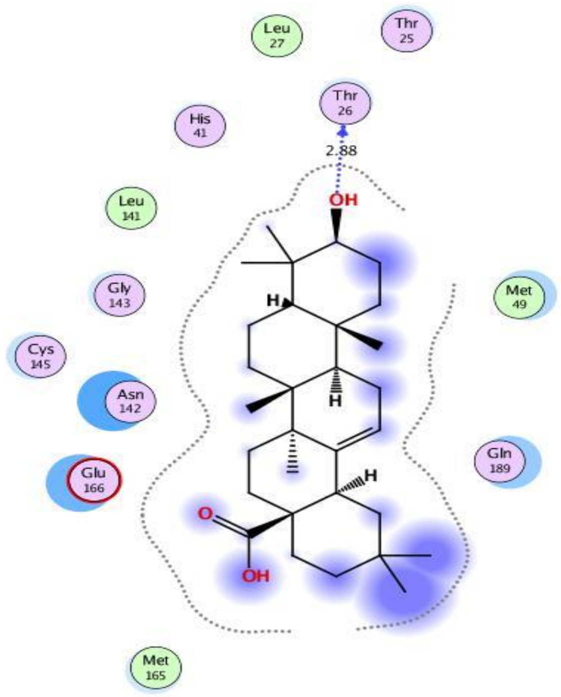
2	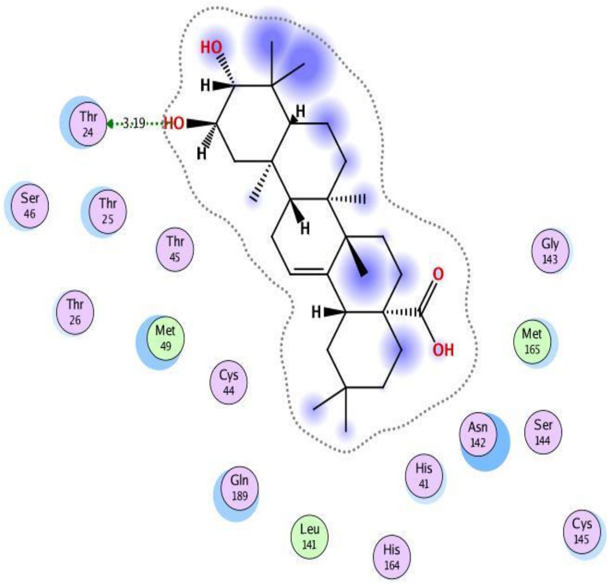

<table>
<tr><td>3</td><td>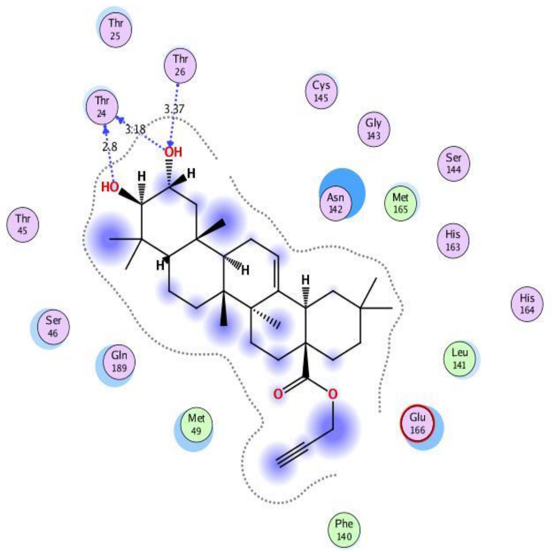
</td></tr>
<tr><td>4</td><td>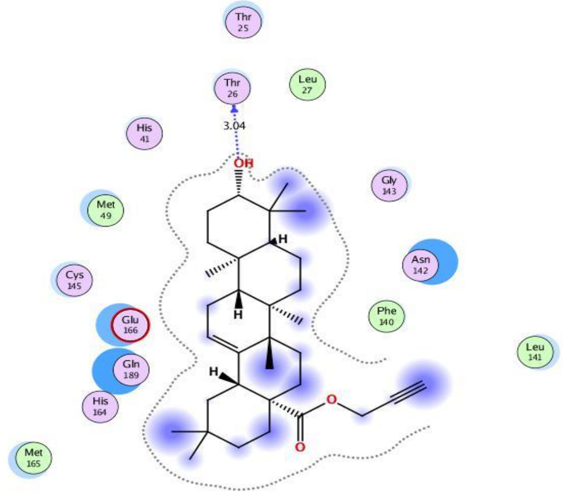
</td></tr>
<tr><td>5</td><td>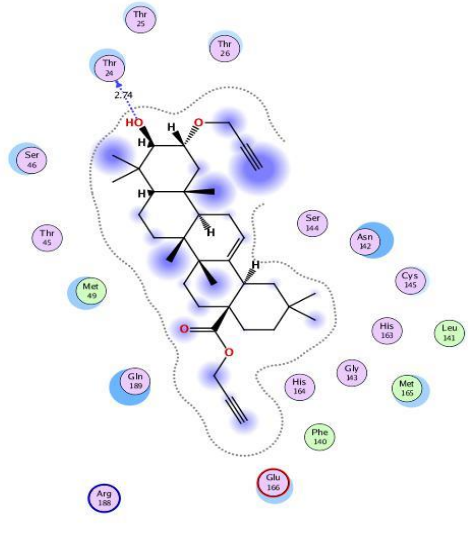
</td></tr>
</table>

6	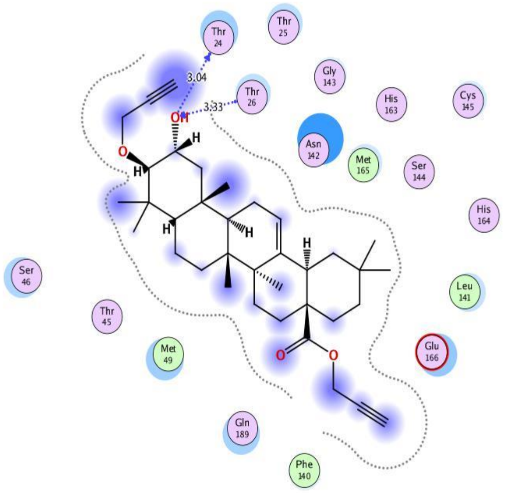 Thr 24 · Thr 25 · 3.04 · Gly 143 · Thr 26 · 3.33 · OH · His 163 · Cys 145 · Asn 142 · O · H · Met 165 · Ser 144 · His 164 · H · H · H · Ser 46 · Leu 141 · Thr 45 · O · O · Glu 166 · Met 49 · Gln 189 · Phe 140
7	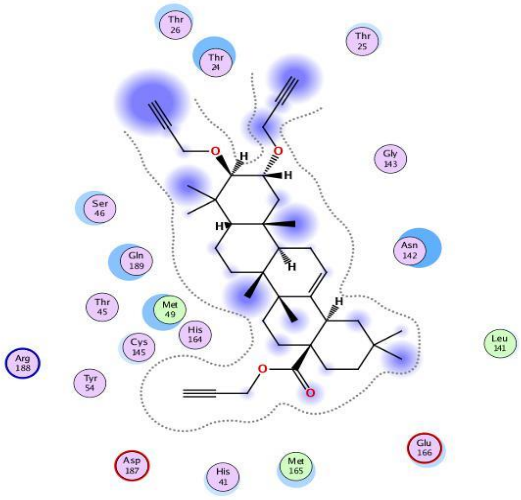 Thr 26 · Thr 24 · Thr 25 · O · O · H · H · Gly 143 · Ser 46 · H · Asn 142 · Gln 189 · H · Thr 45 · Met 49 · H · His 164 · Cys 145 · Leu 141 · Arg 188 · Tyr 54 · O · O · Asp 187 · His 41 · Met 165 · Glu 166
8	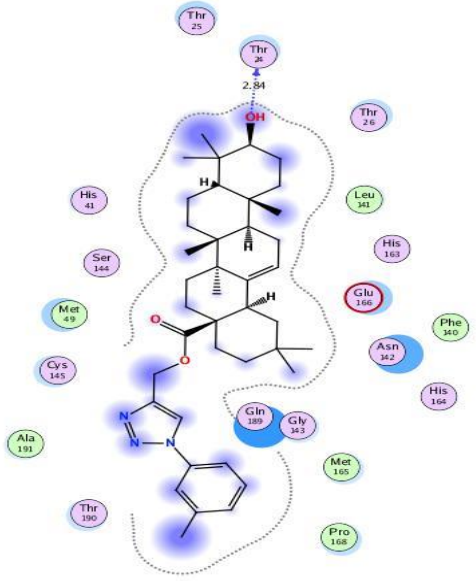 Thr 25 · Thr 24 · 2.84 · OH · Thr 26 · His 41 · H · Leu 141 · His 163 · H · Ser 144 · Glu 166 · H · Met 49 · O · Phe 140 · Asn 142 · O · Cys 145 · His 164 · N · N · N · Gln 189 · Gly 143 · Ala 191 · Met 165 · Thr 190 · Pro 168

9

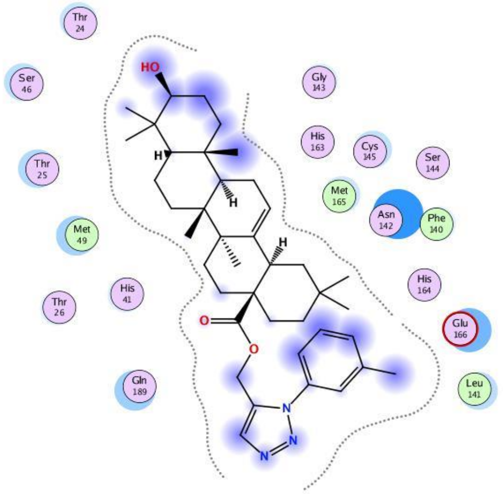
Thr 24
Ser 46
Thr 25
Met 49
Thr 26
His 41
Gln 189
Gly 143
His 163
Cys 145
Ser 144
Met 165
Asn 142
Phe 140
His 164
Glu 166
Leu 141

10

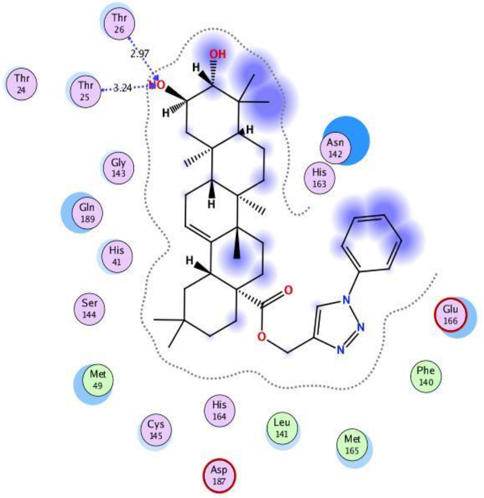
Thr 26
2.97
Thr 24
Thr 25
3.24
Asn 142
His 163
Gly 143
Gln 189
His 41
Ser 144
Glu 166
Phe 140
Met 49
Cys 145
His 164
Leu 141
Met 165
Asp 187

11

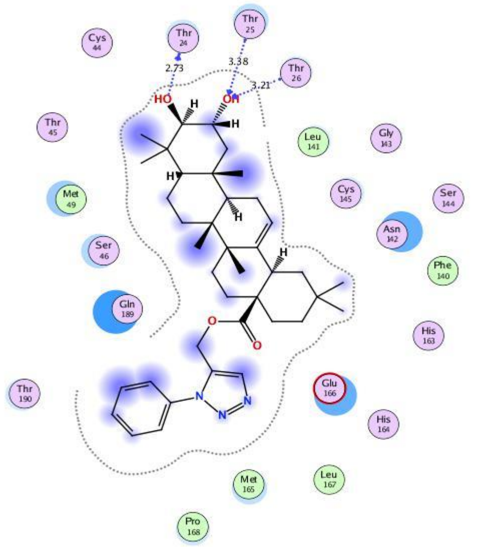
Cys 44
Thr 24
Thr 25
2.73
3.38
Thr 26
3.21
Thr 45
Leu 141
Gly 143
Met 49
Cys 145
Ser 144
Ser 46
Asn 142
Phe 140
Gln 189
His 163
Thr 190
Glu 166
His 164
Met 165
Leu 167
Pro 168

12	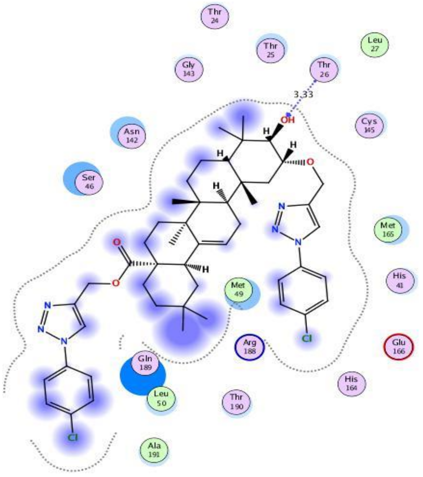
13	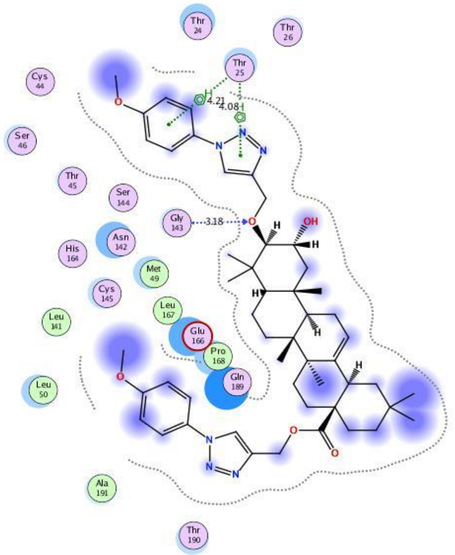
14	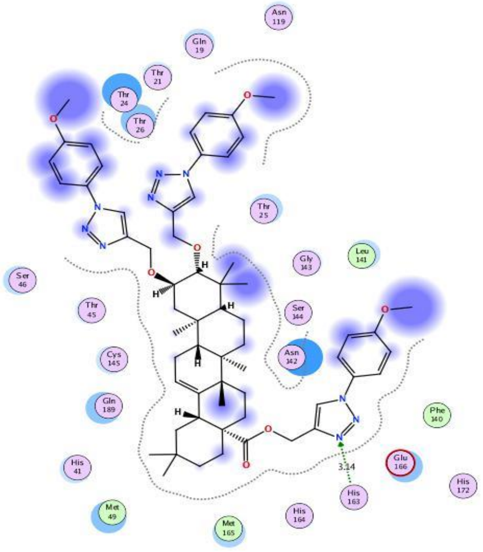

15

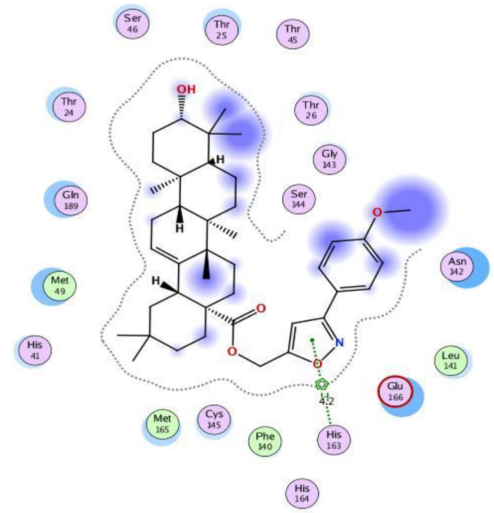
Ser 46
Thr 25
Thr 45
Thr 24
Thr 26
Gly 143
Gln 189
Ser 144
Asn 142
Met 49
His 41
Leu 141
Glu 166
4.2
Met 165
Cys 145
Phe 140
His 163
His 164
OH
H
O
N

16

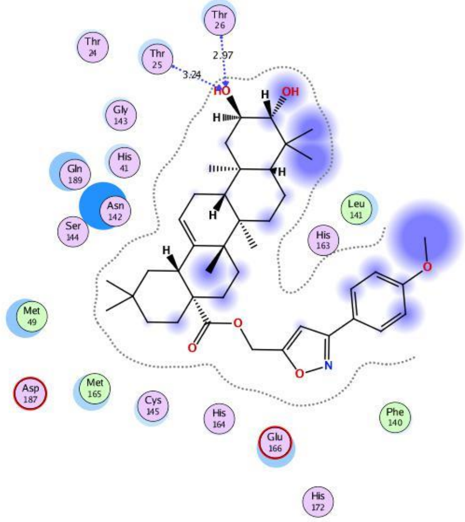
Thr 26
Thr 24
Thr 25
2.97
3.24
HO
OH
H
Gly 143
His 41
Gln 189
Asn 142
Ser 144
Leu 141
His 163
Met 49
Asp 187
Met 165
Cys 145
His 164
Glu 166
Phe 140
His 172
O
N

17

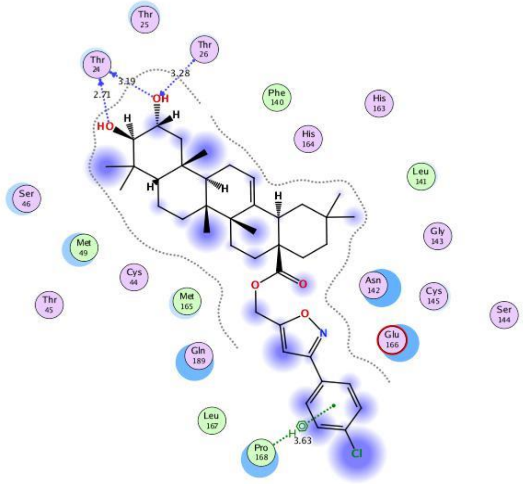
Thr 25
Thr 26
Thr 24
3.19
3.28
2.71
HO
OH
H
Phe 140
His 163
His 164
Leu 141
Ser 46
Gly 143
Met 49
Cys 44
Asn 142
Cys 145
Ser 144
Thr 45
Met 165
Glu 166
Gln 189
Leu 167
Pro 168
3.63
O
N
Cl

18	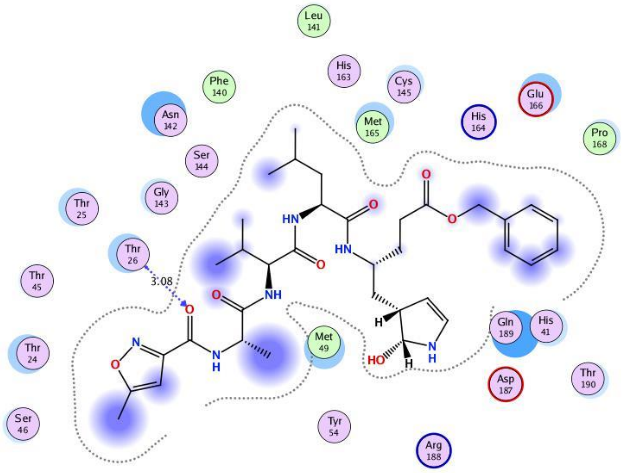Leu 141<br>His 163<br>Phe 140<br>Cys 145<br>Glu 166<br>Asn 142<br>Met 165<br>His 164<br>Pro 168<br>Ser 144<br>Thr 25<br>Gly 143<br>Thr 26<br>Thr 45<br>3.08<br>Gln 189<br>His 41<br>Met 49<br>Thr 24<br>Asp 187<br>Thr 190<br>Ser 46<br>Tyr 54<br>Arg 188
